# Development of CRISPR/Cas13-based analytical tools to study RNA–protein interactions

**DOI:** 10.1093/nar/gkag928

**Published:** 2026-10-13

**Authors:** Keita Yamamoto, Shiori Shikata, Yangying Hao, Takuya Tomita, Rina Kanai, Els Verhoeyen, Yasushi Saeki, Susumu Goyama

**Affiliations:** Department of Computational Biology and Medical Sciences, Graduate School of Frontier Sciences, The University of Tokyo, Tokyo, 108-8639,Japan; Department of Computational Biology and Medical Sciences, Graduate School of Frontier Sciences, The University of Tokyo, Tokyo, 108-8639,Japan; Department of Computational Biology and Medical Sciences, Graduate School of Frontier Sciences, The University of Tokyo, Tokyo, 108-8639,Japan; Division of Protein Metabolism, The Institute of Medical Science, The University of Tokyo, Tokyo, 108-8639,Japan; Protein Metabolism Project, Tokyo Metropolitan Institute of Medical Science, Tokyo, 156-8506,Japan; Department of Computational Biology and Medical Sciences, Graduate School of Frontier Sciences, The University of Tokyo, Tokyo, 108-8639,Japan; CIRI-International Center for Infectiology Research, Inserm, U1111, Université Claude Bernard Lyon 1, CNRS, UMR5308, Ecole Normale Supérieure de Lyon, Université Lyon, 69007,Lyon, France; Université Côte d’Azur, INSERM, C3M, 06204 Nice, France; Division of Protein Metabolism, The Institute of Medical Science, The University of Tokyo, Tokyo, 108-8639,Japan; Protein Metabolism Project, Tokyo Metropolitan Institute of Medical Science, Tokyo, 156-8506,Japan; Department of Computational Biology and Medical Sciences, Graduate School of Frontier Sciences, The University of Tokyo, Tokyo, 108-8639,Japan

## Abstract

RNA–protein interactions (RPIs) are as important as protein–protein interactions (PPIs) for the formation of membraneless organelles (MLOs) and play vital roles in diverse biological processes. Despite remarkable advances in PPI analysis technologies, the development of tools for RPI analysis has lagged behind. To address this challenge, we integrated three established PPI technologies—bimolecular fluorescence complementation (BiFC), NanoBiT, and split-TurboID—with RNA-targeting CRISPR/dCas13. We applied these platforms to paraspeckles, a representative MLO formed through interactions between the long non-coding RNA *NEAT1* and the RNA-binding protein NONO. BiFC-dCas13 functions as a fluorescence-based recorder that enables quantitative detection of *NEAT1*–NONO interaction events. NanoBiT-dCas13 precisely monitors dynamic changes in the *NEAT1*–NONO interaction through a reversible luminescence readout. Split-TurboID-dCas13 induces proximity-dependent biotinylation of proteins surrounding paraspeckles, identifying the N6-methyladenosine reader YTHDC1 as a paraspeckle-associated protein. Furthermore, BiFC-dCas13 was readily reconfigured to detect additional RPI pairs, demonstrating the modularity of the platform. Together, these complementary systems provide versatile approaches for recording, monitoring, and discovering RPI-associated biology and molecular environments.

## Graphical Abstract

**Graphical Abstract:**
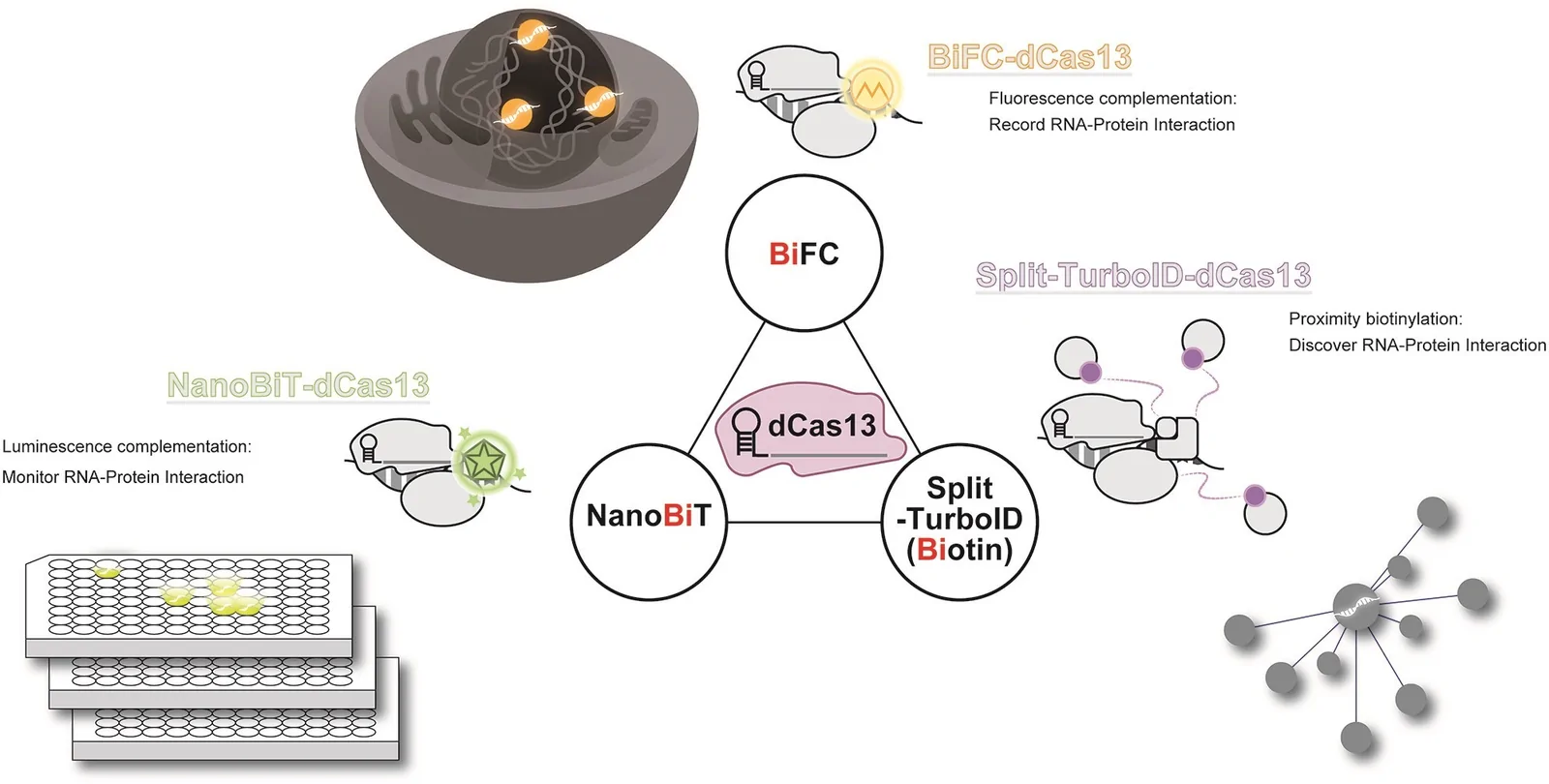

## Introduction

Cells are composed of a variety of molecules, including nucleic acids and proteins. Some of these molecules assemble through liquid–liquid phase separation (LLPS) into biological condensates known as membraneless organelles (MLOs). The past decade has seen a surge in research activity surrounding LLPS and MLOs. Many MLOs are composed of RNA and RNA-binding proteins, highlighting the important role of RNA–protein interactions (RPIs) in MLO formation [1]. Therefore, the development of analytical tools to study RPIs holds immense potential for advancing our understanding of MLOs and their underlying biological functions.

Bimolecular fluorescence complementation (BiFC) is a technology that allows the detection of protein–protein interactions (PPIs) by fluorescence, in which the complementation of two split fluorescent protein fragments is induced by the interaction between proteins of interest [2]. Similarly, the luminescence reporter system based on split luciferase, called “NanoBiT,” is a robust technology for the detection of dynamic PPIs in living cells [3]. Both technologies employ signals that can be detected using tools that are commonly available in a basic laboratory setting, including fluorescence microscopy and microplate readers. Therefore, BiFC and NanoBiT are applicable to a wide range of biomedical research. Proximity labeling, such as BioID, is another major advancement in the field of PPI research in the last decade. Combined with streptavidin (SA)-based affinity purification followed by mass spectrometry analysis, proximity labeling allows us to investigate the PPI networks surrounding MLOs, where the molecular interactions of components are highly fluid, i.e. often transient and dynamic. Split-TurboID is the pair of split fragments of biotin ligase (BL), which is advantageous for the detection of complexes induced by PPIs of two specific proteins of interest. While these technologies are highly applicable in many contexts, their use is currently limited to PPI analysis. Although RPIs are just as important as PPIs in MLOs, versatile analytical tools to investigate specific RPIs in living cells are still lacking.

Cas13 is a CRISPR protein that targets RNA [4] and has been widely used as a highly specific RNA knockdown tool. In addition, catalytically inactive Cas13, referred to as dCas13, has become a powerful tool to study RNA biology. In a previous study, dCas13 combined with a fluorescent protein was established for tracking RNA in living cells [5]. Another study also developed dCas13 combined with BL to detect proteins surrounding specific RNAs [6]. While these technologies have opened new avenues for RNA research in living cells, they were not designed to detect or analyze specific RPIs. To overcome this limitation, we combined several established PPI tools with the RNA-targeting CRISPR/dCas13 and developed novel technologies: BiFC-dCas13, NanoBiT-dCas13, and split-TurboID-dCas13. As a proof-of-concept, we applied these tools to the analysis of paraspeckles, one of the best-known MLOs in the nucleus consisting of RNA (e.g. *NEAT1*) and proteins (e.g. NONO). Our novel CRISPR/dCas13-based RPI analysis platforms enabled fluorescence- and luminescence-based detection of the *NEAT1*–NONO interaction and discovery of the *NEAT1*–NONO-centered molecular environment. Furthermore, we demonstrated the modularity of this framework by extending it beyond the original *NEAT1*–NONO interaction pair to additional RPI systems.

## Materials and methods

### Cell culture

293T cells were cultured in Dulbecco’s modified Eagle’s medium containing 10% fetal bovine serum (FBS) and 1% Penicillin-Streptomycin (Nacalai Tesque). K562 cells were cultured in RPMI-1640 medium supplemented with 10% FBS and 1% Penicillin-Streptomycin (Nacalai Tesque). Cells were cultured in humidified incubators at 37°C in an atmosphere of 5% CO_2_.

### Plasmids

All plasmids used in this study were cloned by polymerase chain reaction (PCR) amplification and HiFi DNA assembly methods. For the construction of dCas13 fusion genes, dCas13 and dCas13-dsRBD were used as backbone vectors [6, 7]. For the construction of NONO fusion genes, pMYs-IN NONO was used as the backbone vector [8]. For the construction of YTHDC1 fusion genes, pcDNA3-FLAG-HA-hYTHDC1 was used as the backbone vector (Addgene plasmid # 85167) [9]. DNA sequences of VN155/VC155 [2], LgBiT/SmBiT (Promega) [3], and TbN73/TbC74 [10] were PCR amplified using Phusion High-Fidelity DNA Polymerase (New England Biolabs) or KOD FX Neo (TOYOBO) and assembled into an enzymatically restricted backbone vector using the NEB HiFi DNA Assembly Kit (New England Biolabs). To generate the D2-destabilized BiFC-dCas13 variant, a D2 degradation domain [11] was fused to the C-terminus of VC155 fragment with the aim of shortening the half-life of the reconstituted fluorescent protein. For CRISPR/Cas9 knock-in, 400 bp of left and right homology arms (HAs) of *YTHDC1*, flanked by a guide RNA targeting sequence and protospacer adjacent motif (PAM), were designed and synthesized using GeneArt Gene Synthesis (Thermo Fisher Scientific). The mCherry sequence was assembled between the two HAs by PCR amplification and HiFi DNA assembly methods. NEB 5-alpha competent *Escherichia coli* (New England Biolabs) was used for plasmid propagation. For crRNA production or guide RNA construction, annealed oligos were inserted into a crRNA backbone vector [7] or lentiGuide-Puro vector [12]. For the construction of dual crRNA arrays, complementary oligonucleotides encoding two different spacer sequences separated by a direct repeat (DR) sequence were annealed and ligated into the crRNA expression vector. For the construction of triple crRNA arrays, a fragment containing an additional DR36 and spacer sequence was PCR-amplified and cloned into the linearized dual crRNA array plasmid using HiFi DNA assembly. The dual and triple crRNA arrays contained crNEAT1 #2-#1 and crNEAT1 #2-#1-#5, respectively. The full sequences of the tandem crRNA constructs are provided in Supplementary Table S1. crRNA sequences were designed with Cas13design (https://cas13design.nygenome.org/). sgRNA sequences were designed with CRISPick (https://portals.broadinstitute.org/gppx/crispick/public). The sequences of the crRNAs and sgRNAs used in this study are described in the Supplementary Table S1.

pXR002: EF1a-dCasRx-2A-EGFP (Addgene plasmid # 109050; http://n2t.net/addgene:109050; RRID:Addgene_109050) and pXR003: CasRx gRNA cloning backbone (Addgene plasmid # 109053; http://n2t.net/addgene:109053; RRID:Addgene_109053) were gifts from Patrick Hsu. 3xHA-TurboID-NLS_pCDNA3 was a gift from Alice Ting (Addgene plasmid # 107171; http://n2t.net/addgene:107171; RRID:Addgene_107171). lentiCas9-Blast (Addgene plasmid # 52962; http://n2t.net/addgene:52962; RRID:Addgene_52962) and lentiGuide-Puro (Addgene plasmid # 52963; http://n2t.net/addgene:52963; RRID:Addgene_52963) were gifts from Feng Zhang. Lentiviral packaging vectors [(pMD2.G # 12259; http://n2t.net/addgene:12259; RRID:Addgene_12259) and (psPAX2 # 12260; http://n2t.net/addgene:12260; RRID:Addgene_12260)] were gifts from Didier Trono. pDGM6 was a gift from David Russell (Addgene plasmid # 110660; http://n2t.net/addgene:110660; RRID:Addgene_110660). BaEVRless [13] was a gift from Els Verhoeyen.

### Transient transfection and virus (or virus-like particle) production

For transient transfection of BiFC-dCas13, NanoBiT-dCas13, and split-TurboID-dCas13, 293T cells at ∼50% confluence were transfected with DNA plasmids using polyethyleneimine (PEI) or Lipofectamine 3000 (Thermo Fisher Scientific), and subjected to further assays 1–2 days after transfection. The mixing ratio of the transfected DNA was dCas13:NONO:crRNA = 1:3:2 (weight ratio), unless otherwise noted.

For lentivirus, adeno-associated virus (AAV) and MMLV virus-like particle (VLP) production [14], 293T cells at ∼80% confluence were transfected with DNA plasmids using the calcium-phosphate method. Lentiviruses were produced by transient transfection of 293T cells with viral plasmids together with lentiviral packaging plasmids (pMD2.G and psPAX2). AAVs were produced by transient transfection of 293T cells with viral plasmids together with AAV serotype 6 packaging plasmids (pDGM6). Cas9-MMLV VLPs were produced by transient transfection of 293T cells with MMLV gag-Cas9 plasmids and sgRNA plasmids together with MMLV gag-pol and envelope expressing plasmids (VSVg and BaEVRless [15, 16]). All virus (VLP) particles were harvested from the supernatant 48–72 h post-transfection and filtered through a 0.45 μm PVDF membrane filter. For Cas9-MMLV VLP concentration, 1/3 volume of Lenti-X Concentrator (Takara) was added to the supernatant, incubated overnight at 4°C, and pelleted by centrifugation at 1500 × *g* for 45 min at 4°C. The VLP pellet was resuspended in Opti-MEM to achieve a 10-fold concentration. Viruses (or VLPs) were stored at −80°C and thawed immediately before use.

### Gene knockout using CRISPR/Cas9

For *YTHDC1* and *YTHDC2* depletion, K562 cells were transduced with Cas9 (coexpressing blasticidin S deaminase) using lentiviruses produced as described earlier. After 1 week of blasticidin selection, K562/Cas9 cells were transduced with guide RNAs (coexpressing a puromycin resistance gene). After 3 days of puromycin selection, the cells were subjected to the RNA or protein extraction, or RNA-FISH analysis. The sequences of the sgRNAs used in this study are described in the Supplementary Table S1.

### Gene knock-in using CRISPR/Cas9

For gene editing (knock-in) of the K562 genome, donor DNA of mNG211 was prepared as ssODN (IDT). Donor DNA of mCherry was prepared as an AAV-producing plasmid as described earlier. Donor DNA was transfected into K562 cells using Lipofectamine 3000 (Thermo Fisher Scientific) or rAAV6 viral transduction, followed by target-site specific Cas9 VLP to induce homology-directed repair.

The sequences of the sgRNAs used in this study are described in the Supplementary Table S1.

### Live-cell imaging and RNA FISH followed by immunofluorescence

Fluorescence images were captured using an EVOS imaging system (Invitrogen) or a confocal microscope system (A1Rsi [Nikon] or IX73 [Olympus] equipped with a spinning disk confocal unit CSU-W1 [Yokogawa]). Images were analyzed using NIS-Elements software or ImageJ Fiji image analysis software. For sample preparation, 293T cells cultured on round glass slides or K562 cells cultured on poly-L-lysine-coated glass slides were used. RNA FISH was performed according to the manufacturer’s instructions. Briefly, cells were fixed with 4% paraformaldehyde and permeabilized with 70% ethanol. The cells were then hybridized with Stellaris FISH probes targeting the human *NEAT1* middle segment with Quasar 570 dye, and stained with anti-NONO antibodies, followed by labeling with Alexa Fluor 488-conjugated anti-mouse (Invitrogen, A-11029) or Alexa Fluor 633-conjugated anti-rabbit (Invitrogen, A-21086) antibodies. Cell nuclei were visualized with DAPI.

### Image analysis

Quantitative image analyses were performed using ImageJ Fiji. For quantification of BiFC-positive cells, cells in which the mVenus-positive area exceeded 10% of the DAPI-positive nuclear area were classified as BiFC-positive. *NEAT1*-positive regions were defined from thresholded *NEAT1* RNA-FISH images, and mVenus fluorescence intensity within these regions was measured. The area, circularity, and Feret diameter of *NEAT1*-positive foci were quantified using the particle analysis function in ImageJ Fiji. The numbers of cells and independent experiments analyzed are provided in the corresponding figure legends.

### Flow cytometry

For quantification of BiFC-dCas13, mVenus fluorescence intensity was analyzed using a CytoFLEX LX flow cytometer (Beckman Coulter) with CytExpert software. To isolate genome-edited K562 cells, mNeonGreen-positive, mCherry-positive, and mNeonGreen/mCherry double-positive cells were sorted using a FACSAria cell sorter (BD Biosciences) with FACSDiva software.

For analysis of transiently transfected 293T cells, cells expressing the BiFC-dCas13 system, including dCas13 fusion proteins carrying VN155 or VC155, together with NONO fusion proteins carrying the complementary Venus fragment and the indicated crRNA, were detached by trypsinization, collected by centrifugation, and resuspended in phosphate buffered saline (PBS) containing 2% FBS. Gene-edited K562 cells were collected by centrifugation and similarly resuspended in PBS containing 2% FBS. DAPI was used to exclude dead cells.

In the NONO-VC155- and VN155-NONO-expressing vectors used for the BiFC-dCas13 assay, the NONO fusion coding sequence was followed by an internal ribosome entry site and an NGFR reporter gene. After trypsinization, cells were stained with an APC-conjugated anti-NGFR antibody (BioLegend, 345107) for 30 min at 4°C in the dark. DAPI-negative, NGFR-positive cells were selectively gated, and mVenus fluorescence intensity was quantified within this population.

All flow cytometry data were analyzed using FlowJo software (BD Biosciences). The gating strategies are shown in Supplementary Fig. S1.

### Luciferase assay

NanoBiT PPI luminescence was measured using the Nano-Glo Live Cell Assay System (Promega) according to the manufacturer’s instructions. Briefly, 293T cells were seeded in 96-well culture plates at a density of 10 000 cells per well. At 18 h after seeding, the cells were transfected with the NanoBiT-dCas13 system using PEI. Nano-Glo Live Cell Reagent was added 2–3 days after transfection, and cells were assayed for luciferase activity using the luciferase assay system (Promega) and a luminometer (BMG LABTECH, FLUOstar OPTIMA).

### Quantitative RT-PCR

RNA was extracted using TRIzol and reverse transcribed using High-Capacity complementary DNA (cDNA) Reverse Transcription Kits (Applied Biosystems, 4368814) according to the manufacturer’s protocol. cDNA was then subjected to quantitative reverse transcriptase-polymerase chain reaction (RT-PCR) using a SYBR Select Master Mix (Applied Biosystems) with a StepOnePlus System (Applied Biosystems). The sequences of the primers used for qRT-PCR in this study are described in the Supplementary Table S1.

### Biotin pulldown assay

293T cells were transiently transfected with the split-TurboID-dCas13 system using PEI. Biotinylation was induced 48 h after transfection for the indicated time, and cells were lysed in total cell lysis buffer [50 mM Tris–HCl, pH 7.5, 1 mM ethylenediaminetetraacetic acid (EDTA), 150 mM NaCl, 1% Triton X-100, 2 mM phenylmethylsulfonyl fluoride (PMSF), and 1 mM dithiothreitol (DTT), protease inhibitor cocktail (Sigma)]. Sample lysates were incubated with Dynabeads M-280 Streptavidin (Invitrogen) for 30 min at room temperature. Precipitates were washed three times with cell lysis buffer and eluted in Laemmli sample buffer (Bio-Rad). Samples were subjected to sodium dodecyl sulfate–polyacrylamide gel electrophoresis (SDS–PAGE) followed by gel staining or western blot. For Oriole staining, the gel was stained with Oriole fluorescent gel stain (Bio-Rad) for >2 h at room temperature and visualized. For western blotting, transferred proteins were stained with an anti-biotin antibody (Santa Cruz Biotechnology). Signals were detected with ECL Western Blotting Substrate (Promega), and immunoreactive bands were visualized with a LAS-4000 Luminescent Image Analyzer (FUJIFILM). Band intensities were analyzed using LabWorks version 4.5 software (UVP, LLC).

### Mass-spectrometric analyses

The LC-MS/MS analysis was performed essentially as described previously with minor modifications [17]. Analytes were separated by SDS–PAGE with a short run (1 cm) and stained with Bio-Safe Coomassie (Bio-Rad). The gel pieces were washed with 50 mM ammonium bicarbonate (AMBC)/30% acetonitrile (ACN) for 30 min and with 50 mM AMBC/50% ACN for 30 min. Proteins were reduced in 10 mM DTT/50 mM AMBC for 15 min at 56°C and alkylated in 55 mM iodoacetamide (IAA)/50 mM AMBC for 45 min at RT in the shade. The gel pieces were dehydrated in 50% ACN/50 mM AMBC for 10 min and then in 100% ACN for 5 min, followed by in-gel tryptic digestion with 20 ng/μl Trypsin Gold (Promega) in 50 mM AMBC/5% ACN at 37°C for 16 h. The digested peptides were extracted with 0.1% trifluoroacetic acid (TFA)/50% ACN, and 0.1% TFA/70% ACN, followed by concentration using a vacuum centrifuge.

The peptides were resuspended in 0.1% TFA and analyzed using an Easy-nLC1200 (Thermo Fisher Scientific) connected online to an Orbitrap Fusion LUMOS (Thermo Fisher Scientific) with a nanoelectrospray ion source (Thermo Fisher Scientific). Peptide samples were loaded onto a C18 analytical column (IonOpticks, Aurora Series Emitter Column, AUR2-25075C18A 25 cm × 75 μm 1.6 μm FSC C18 with nanoZero fitting) and separated using a 150-min gradient (solvent A, 0.1% FA; solvent B, 80% ACN/0.1% FA). For data-dependent acquisition of MS/MS spectra, the most intense ions (Cycle Time: 3 s) with charge state +2 to +7 were selected for fragmentation by higher-energy collisional dissociation with a normalized collision energy of 30 and detected using Orbitrap. The resolution, duration of dynamic exclusion, and isolation window were set to 120 000, 15 s, and 1.6 m/z, respectively.

The data were analyzed using a Sequest HT search program in Proteome Discoverer 2.4 (Thermo Fisher Scientific). MS/MS spectra were searched against the SwissProt-reviewed *Homo sapiens* reference proteome (UniProt v2017-10-25). Intensity-based label-free protein quantification was performed using a Precursor Ions Quantifier node in Proteome Discoverer 2.4. The mass tolerances for the precursor and fragment were 10 ppm and 0.02 Da, respectively. Maximum missed cleavage sites of trypsin were set to 2. Oxidation (Met), biotinylation (Lys), deamidation (Gln, Asn), and acetylation (protein N terminus) were selected as variable modifications. Peptide identification was filtered at FDR < 0.01.

### RNA-IP followed by quantitative RT-PCR

293T cells were seeded and transiently transfected with either a mock vector or a FLAG-YTHDC1 expression plasmid using PEI at ∼50% confluence, and cultured for an additional 48 h. Cells were harvested after washing with PBS and resuspended in 2 ml PBS. Nuclear isolation was performed by adding 2 ml nuclear isolation buffer [1.28 M sucrose, 40 mM Tris–HCl (pH 7.5), 20 mM MgCl_2_, and 4% Triton X-100], together with 6 ml distilled water. Samples were gently rotated at 4°C for 20 min with occasional mixing, and nuclei were collected by centrifugation. The nuclear pellet was resuspended in 1 ml RIP buffer [25 mM Tris–HCl, pH 7.4, 150 mM KCl, 5 mM EDTA, 0.5% NP-40, 0.5 mM DTT, protease inhibitor cocktail (Sigma–Aldrich, P8340), and RNase inhibitor (TaKaRa, 2313A)]. Nuclear lysates were sonicated on ice for 20 s at 40% amplitude and centrifuged. The resulting supernatant was used for RNA immunoprecipitation, and an aliquot was retained as the input fraction. For each immunoprecipitation, 25 µl of Protein G Dynabeads (Thermo Fisher) were washed with IP buffer and incubated with an anti-FLAG antibody (Sigma–Aldrich, F3165) for 10 min at room temperature with rotation. Antibody-bound beads were washed once and then incubated with the nuclear lysate for 30 min at room temperature with rotation. The beads were washed three times with RIP buffer. RNA was extracted from the immunoprecipitated material using TRIzol reagent and subjected to RT-qPCR. An aliquot of each input and immunoprecipitated fraction was subjected to SDS–PAGE and immunoblotting to confirm FLAG-YTHDC1 expression and immunoprecipitation efficiency. FLAG-YTHDC1 and endogenous NONO were detected using the indicated antibodies.

Because *NEAT1* was not reliably detected in the mock immunoprecipitation fraction, normalization to the corresponding input fraction was not performed. Instead, raw threshold cycle (Ct) values from mock input, FLAG-YTHDC1 input, mock immunoprecipitation, and FLAG-YTHDC1 immunoprecipitation samples were presented. Primer sequences are provided in Supplementary Table S1.

### Co-IP and immunoblotting

293T cells were seeded and transiently transfected with FLAG-YTHDC1 and/or Myc-NONO expression plasmids using PEI at ∼50% confluence, and cultured for an additional 48 h. Cells were harvested, washed once with PBS, and lysed in IP buffer [50 mM Tris–HCl, pH 7.5, 1 mM EDTA, 150 mM NaCl, 1% Triton X-100, 50 µM NaF, 2 mM PMSF, 2 mM Na3VO4, 0.1% SDS, and protease inhibitor cocktail (Sigma–Aldrich, P8340)]. Lysates were vortexed for 10 s and clarified by centrifugation. An aliquot of each clarified lysate was retained as the input fraction.

For immunoprecipitation, 25 µl of Protein G Dynabeads were washed with IP buffer and incubated with an anti-FLAG antibody (Sigma–Aldrich, F3165) for 10 min at room temperature with rotation. Antibody-bound beads were then incubated with the clarified cell lysates for 30 min at room temperature with rotation. The beads were washed three times with 500 µl IP buffer and resuspended in 50 µl 2× SDS sample buffer. Immunoprecipitated proteins were eluted by heating at 95°C for 10 min and subjected to SDS–PAGE followed by immunoblotting with the indicated antibodies.

### AlphaFold structural modeling

Structural models of the BiFC-dCas13 variants were generated using AlphaFold2 [18], whereas models of the BiFC-dCas13-crRNA-target RNA complexes were generated using the AlphaFold3 server [19]. For AlphaFold2 analysis, the amino acid sequence of each indicated BiFC-dCas13 variant was provided as input, and five generated models were used for further analysis. For AlphaFold3 analysis, the amino acid sequence of the indicated BiFC-dCas13 variant, the full mature cr*NEAT1*#1 sequence containing the DR and spacer, and the corresponding *NEAT1* target RNA sequence complementary to the spacer were provided as input, and the top-ranked model from each AlphaFold3 prediction was used for subsequent analysis. The resulting models were visually compared to evaluate the overall structure of the BiFC-dCas13 variants and the predicted positioning of the dsRNA-binding domain relative to the dCas13-bound crRNA and target RNA. Molecular structures were inspected and rendered using Mol* or PyMOL. Predicted aligned error (PAE) matrices were extracted from the full-data JSON files generated by AlphaFold3 and visualized using custom Python scripts with NumPy and Matplotlib. PAE values were displayed on a common scale ranging from 0 to 30 Å, and boundaries between protein and RNA chains were determined from the token_chain_ids information contained in the AlphaFold3 output.

### Quantification and statistical analysis

Statistical significance was determined by Fisher’s exact test, unpaired two-tailed *t*-test, or one-way analysis of variance (ANOVA) with post hoc test, testing for normal distribution and equal variance, unless otherwise indicated. Sample sizes, experimental replicates, and specific statistical tests used are described in the figure legends. Data were presented as means using GraphPad Prism 9 software. Error bars represent SD.

### Declaration of generative AI and AI-assisted technologies in the manuscript preparation process

During the preparation of this work, the authors used ChatGPT to improve the readability and language of the manuscript and to assist in writing analytical code. After using this tool, the authors reviewed and edited the content as needed and take full responsibility for the content of the published article.

## Results

### Generation of the BiFC-dCas13 system

First, we sought to establish a BiFC-dCas13 system as a fluorescent reporter of *NEAT1*–NONO interactions. In this system, the split mVenus is regenerated when *NEAT1*-targeting dCas13 interacts with NONO (Fig. 1A). We chose CasRx for the development of BiFC-dCas13 because CasRx has been shown to have the highest knockdown efficiency among Cas13 orthologs, indicating that CasRx has the strongest RNA binding ability [7]. We designed the first generation BiFC-dCas13 as a simple fusion protein, with catalytically dead CasRx (dCas13d) fused directly to an N-terminal fragment of Venus split at amino acid residue 155 (VN155) at the C-terminus (Fig. 1B). As a complementary partner of the Venus fragment, a C-terminal fragment of Venus split at amino acid residue 155 (VC155) was fused to NONO, also at the C-terminus. We transfected these constructs (dCas13-VN155, crRNA, and NONO-VC155) of the BiFC-dCas13 system (BiFC-dCas13_v1) into 293T cells. Live-cell imaging of 293T cells transfected with BiFC-dCas13_v1 visualized mVenus fluorescent foci with cr*NEAT1*, which were assumed to be paraspeckles (Fig. 1C). However, the mVenus signal was also detected with crNT (Fig. 1C and D), suggesting the presence of non-specific binding of dCasRx to RNA, as shown in a previous report [5].

**Figure 1.**
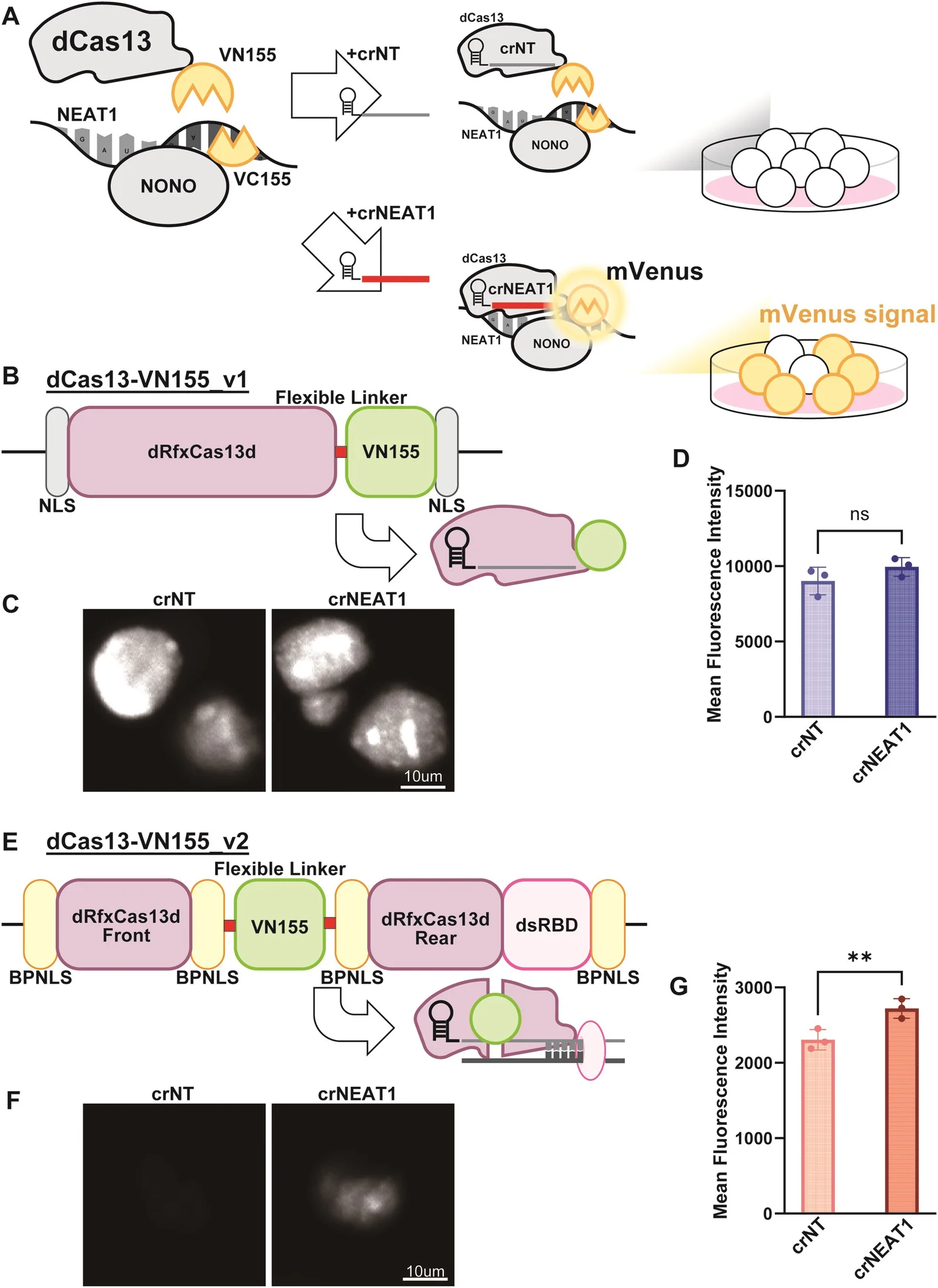
Structural improvement of the BiFC-dCas13 system. (**A**) Schematic of BiFC-dCas13. VN155 and VC155 are fused to dCas13d and a protein of interest (POI; NONO in this study), respectively. dCas13d-VN155 is directed to an RNA of interest (ROI; *NEAT1* in this study). BiFC complementation is induced by a crRNA complementary to the ROI, resulting in mVenus fluorescence. On the other hand, a non-targeting crRNA (crNT), which is unable to direct dCas13d to the ROI, does not induce BiFC complementation. (**B**) Schematic of dCas13d-VN155 version 1. The VN155 domain was fused directly to dCas13d at its C-terminus. SV40 NLSs were located at both ends of the fusion protein. A flexible linker (GGGGSGGGGS) was located between the dCas13d and VN155 domains. (**C**) Live-cell imaging of the BiFC-dCas13_v1 system combined with crNT or cr*NEAT1*. 293T cells were transfected with BiFC-dCas13_v1 components and crRNAs. mVenus fluorescence images were acquired 2 days after transfection using the Nikon A1 confocal microscopic imaging system. Representative mVenus fluorescence images of crNT (left), cr*NEAT1* (right) are shown. Scale bars: 10 μm. (**D**) Mean fluorescence intensity of the BiFC-dCas13_v1 systems are shown as single data points and mean ± SDs. 293T cells were transfected with BiFC-dCas13 components and crNT or cr*NEAT1*. The mVenus fluorescence signal was acquired by flow cytometry 2 days after transfection. (**E**) Schematic of dCas13d-VN155 version 2. In version 2, dCas13d was truncated into anterior (amino acids 1–558) and posterior (amino acids 588–end) domains, and VN155 was inserted between these two fragments. The double-stranded RNA-binding domain (dsRBD) was fused at the C-terminus. The SV40 NLS was replaced by BPNLS. A flexible linker (GGGGSGGGGS) was also placed between the dCas13d and VN155 domains. (**F**) Live-cell imaging of the BiFC-dCas13_v2 system combined with crNT or cr*NEAT1*. 293T cells were transfected with BiFC-dCas13_v2 components and crRNAs. mVenus fluorescence images were acquired 2 days after transfection using the Nikon A1 confocal microscopic imaging system. Representative mVenus fluorescence images of crNT (left), cr*NEAT1* (right) are shown. Scale bars: 10 μm. (**G**) Mean fluorescence intensity of the BiFC-dCas13d_v2 systems are shown as single data points and the mean ± SDs. 293T cells were transfected with BiFC-dCas13 components and crNT or cr*NEAT1*. The mVenus fluorescence signal was acquired by flow cytometry 2 days after transfection.

Since several reports have shown that the Cas13 fusion proteins are most active when they include the effector domain within their structure [20, 21], we next designed a new BiFC-dCas13 containing VN155 or VC155 within the dCas13d peptide sequence (Fig. 1E). Structural modeling using AlphaFold [18] predicted that both dCas13 and VN155 structures are independent, even when VN155 is placed within the dCas13 peptide sequence (Supplementary Fig. S2A and B). In addition, the VN155 domain in the new BiFC-dCas13 is more distant from the center of the fusion protein than in the old version (Supplementary Fig. S3A and B). This indicated that the inserted split-fluorescent fragment can operate with greater steric freedom from the dCas13 domain. We also replaced the SV40 NLS in the BiFC-dCas13_v1 with bipartite NLS sequences (BPNLS) and added a dsRBD, which has been shown to promote the interaction between dCas13 and its target RNA [6]. Of note, structural modeling of the dCas13d–crRNA–target RNA complex indicated that the appended dsRBD was not predicted to grossly perturb the overall dCas13d–crRNA architecture or the crRNA–target RNA interaction interface (Supplementary Fig. S4 and Supplementary Fig. S5A and B). Thus, we developed BiFC-dCas13_v2 as a second generation BiFC-dCas13 system (Fig. 1E). We transfected this BiFC-dCas13_v2 into 293T cells together with crNT or cr*NEAT1* and found strong mVenus fluorescent signals only with cr*NEAT1*, whereas no strong mVenus signals were seen with crNT (Fig. 1F). The mean fluorescence intensity of Venus was also statistically higher in BiFC-dCas13_v2-expressing cells with cr*NEAT1* than in those with crNT (Fig. 1G). Thus, the structurally improved BiFC-dCas13, BiFC-dCas13_v2, achieved a highly specific signal-to-noise (S/N) ratio compared to the old version.

Second, we optimized the effective *NEAT1*-targeting crRNAs from the entire *NEAT1* sequence using a previously developed prediction model [22] (Fig. 2A) and found several effective crRNAs to visualize the *NEAT1*–NONO interaction (Fig. 2B). Cas13 can target different RNA sequences by processing tandemly arrayed pre-crRNAs (spacer and 36-nt DR: DR36) into mature crRNAs (spacer and 30-nt DR: DR30) [7]. Therefore, we investigated whether arrayed crRNAs composed of multiple effective crRNAs targeting different *NEAT1* sequences could improve the S/N ratio in our system (Fig. 2C). As expected, the dual-cr*NEAT1* (#2 + 1) showed higher signal intensity compared to single crRNAs in both flow cytometry analysis (Fig. 2D) and live-cell imaging (Fig. 2E–G). We then constructed a triple-crRNA array targeting *NEAT1* (triple-cr*NEAT1*_#2 + 1 + 5), which showed the highest mVenus reconstitution ability with the BiFC-dCas13_v2 system (Fig. 2H and I; Supplementary Fig. S6).

**Figure 2.**
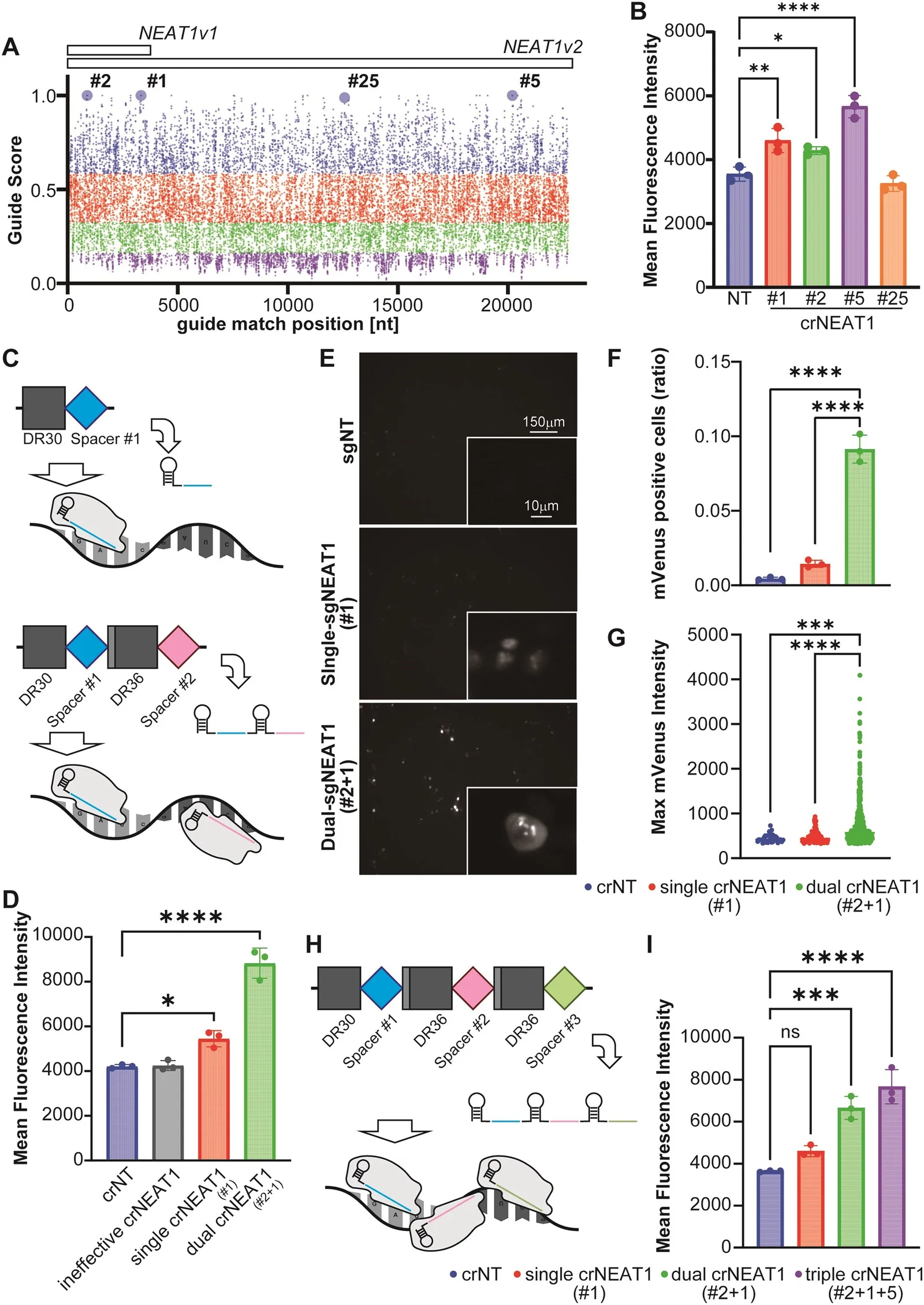
Improvement of the crRNAs used in the BiFC-dCas13 system. (**A**) Guide scores of each crRNA targeting the *NEAT1* transcript position are shown. Guide scores were calculated using Cas13design (https://cas13design.nygenome.org/). Among several transcript variants, the longest isoform of *NEAT1* (ENST00000501122) was selected for guide score screening. Twenty-three-nucleotide crRNAs were designed from the whole transcript sequence and scored according to the Cas13design criteria. Large circles indicate the crRNAs used in the validation assay. (**B**) Mean fluorescence intensity of the version 2 BiFC-dCas13d systems with 5 crRNAs is shown as mean ± SD. 293T cells were transfected with version 2 BiFC-dCas13 components and crNT, or four different types of cr*NEAT1* as indicated in Fig. 2A. The mVenus fluorescence signal was measured 2 days after transfection by flow cytometry. *P < .05; ^****^P < .0001 (one-way ANOVA with Dunnett post hoc test). *n* = 3 for each group. (**C**) Schematic of single- and dual-crRNAs and CRISPR/Cas13 proteins guided to target transcripts. For dual-crRNAs, 2 crRNAs targeting different positions of the same transcript are tandemly placed. Each spacer sequence is preceded by a DR (DR30 for the former and DR36 for the later crRNA) and is processed into mature crRNAs. (**D**) Mean fluorescence intensity of the version 2 BiFC-dCas13 systems with 4 crRNAs is shown as the mean ± SDs. 293T cells were transfected with version 2 BiFC-dCas13 components and crNT, or three different types of cr*NEAT1* (ineffective, #1, and #2 + 1). The mVenus fluorescence signal was measured 2 days after transfection by flow cytometry. *P < .05; ^****^P < .0001 (one-way ANOVA with Dunnett post hoc test). *n* = 3 for each group. (**E**) Live-cell imaging of the BiFC-dCas13_v2 system combined with crNT, single-cr*NEAT1* (#1), and dual-cr*NEAT1* (#2 + 1). 293T cells were transfected with version 2 BiFC-dCas13 components and crRNAs. The mVenus fluorescence images were acquired 2 days after transfection using the EVOS microscope imaging system. Representative mVenus fluorescence images of crNT (top), single-cr*NEAT1* (middle), and dual-cr*NEAT1* (bottom) are shown. Scale bars: 10 and 150 μm. (**F**) Ratios of mVenus-positive cells are shown as individual data points and the mean ± SD. Ratios were calculated by counting the number of mVenus-positive cells (above threshold) and total cells in three separate fields of view from a single representative experiment. Statistical analysis was performed using the field-level measurements. ^****^P < .0001 (one-way ANOVA with Tukey–Kramer post hoc test). (**G**) Maximum mVenus signal intensities of each mVenus-positive cell are shown as individual data points. Signal intensities were measured from 36, 122, and 769 cells, respectively, from a single experiment. Statistical analysis was performed on the cell-level measurements. ***P < .001; ^****^P < .0001 (one-way ANOVA with Tukey–Kramer post hoc test). (**H**) Schematic of the triple-crRNA and CRISPR/Cas13 proteins guided to target transcripts. For triple-crRNA, three crRNAs targeting different positions of the same transcript are tandemly placed. Each spacer sequence is preceded by a DR (DR30 for the first and DR36 for the middle and last crRNAs) and is processed into mature crRNAs. (**I**) Mean fluorescence intensity of the BiFC-dCas13d_v2 system with the indicated crRNAs is shown as the mean ± SDs. 293T cells were transfected with version 2 BiFC-dCas13d components and crNT, or three different types of cr*NEAT1* (#1, #2 + 1, and #2 + 1 + 5). The mVenus fluorescence signal was measured 2 days after transfection by flow cytometry. ***P < .001; ^****^P < .0001 (one-way ANOVA with Dunnett post hoc test). *n* = 3 for each group.

Third, we generated multiple fusion constructs of dCas13 and NONO combined with split mVenus (VN155 or VC155) at the N- or C-terminus to find the best combination with the highest S/N ratio (Supplementary Fig. S7A). All pairs of BiFC-dCas13 and BiFC-NONO showed complementation of the fluorescent protein when cotransfected with *NEAT1*-targeting crRNA in 293T cells. Among them, the combination of dCas13 with VC155 (dCas13-VC155) and NONO with VN155 at its N-terminus (VN155-NONO) showed the highest S/N ratio, as indicated by the low mVenus signal in the crNT control group and the highest mVenus signal in the cr*NEAT1* group (Supplementary Fig. S7B). Live-cell imaging also demonstrated that 293T cells transfected with dCas13-VC155, VN155-NONO, and cr*NEAT1* showed the strongest signal intensity (Supplementary Fig. S7C and D), indicating that dCas13-VC155 and VN155-NONO is the best combination. With these improvements, we have successfully established the third-generation BiFC-dCas13 (BiFC-dCas13_v3; Supplementary Fig. S8).

Finally, we optimized the stoichiometry of the components of the BiFC-dCas13 system. Transfection of dCas13, NONO, and crRNA with a ratio of 1:3:2 (weight ratio) resulted in the highest mVenus expression when cr*NEAT1* was used, while there was no significant change in mVenus expression when crNT was used as a control (Supplementary Fig. S9).

### BiFC-dCas13 successfully records *NEAT1*–NONO interactions in paraspeckles

To evaluate the performance of the optimized BiFC-dCas13 system, we quantified mVenus reconstitution by flow cytometry. Robust fluorescence signals were detected in cells expressing *NEAT1*-targeting crRNAs, whereas only minimal signals were observed with non-targeting controls (Fig. 3A). Consistent with these findings, fluorescence microscopy revealed prominent nuclear mVenus accumulation selectively in the cr*NEAT1* group (Fig. 3B and C). Quantitative image analyses further supported the association between BiFC signal accumulation and *NEAT1*-positive regions (Fig. 3C and D; Supplementary Fig. S10A). Examination of mVenus fluorescence patterns revealed heterogeneous signal distributions among mVenus-positive cells (Fig. 3C). While a subset of cells displayed punctate fluorescence overlapping with *NEAT1*-positive nuclear foci, others exhibited broader nuclear fluorescence extending beyond *NEAT1* foci (Supplementary Fig. S10B). This broader distribution likely reflects the irreversible nature of BiFC complementation, whereby reconstituted fluorophores persist and accumulate despite subsequent redistribution of the original interaction partners. To investigate whether reducing fluorophore stability could improve spatial confinement, we generated a D2-destabilized BiFC-dCas13 variant [11] (Supplementary Fig. S11A). Although this modification modestly reduced diffuse fluorescence accumulation, substantial nuclear fluorescence remained detectable (Supplementary Fig. S11B), indicating that fluorophore destabilization alone was insufficient to overcome the intrinsic irreversibility of BiFC complementation.

**Figure 3.**
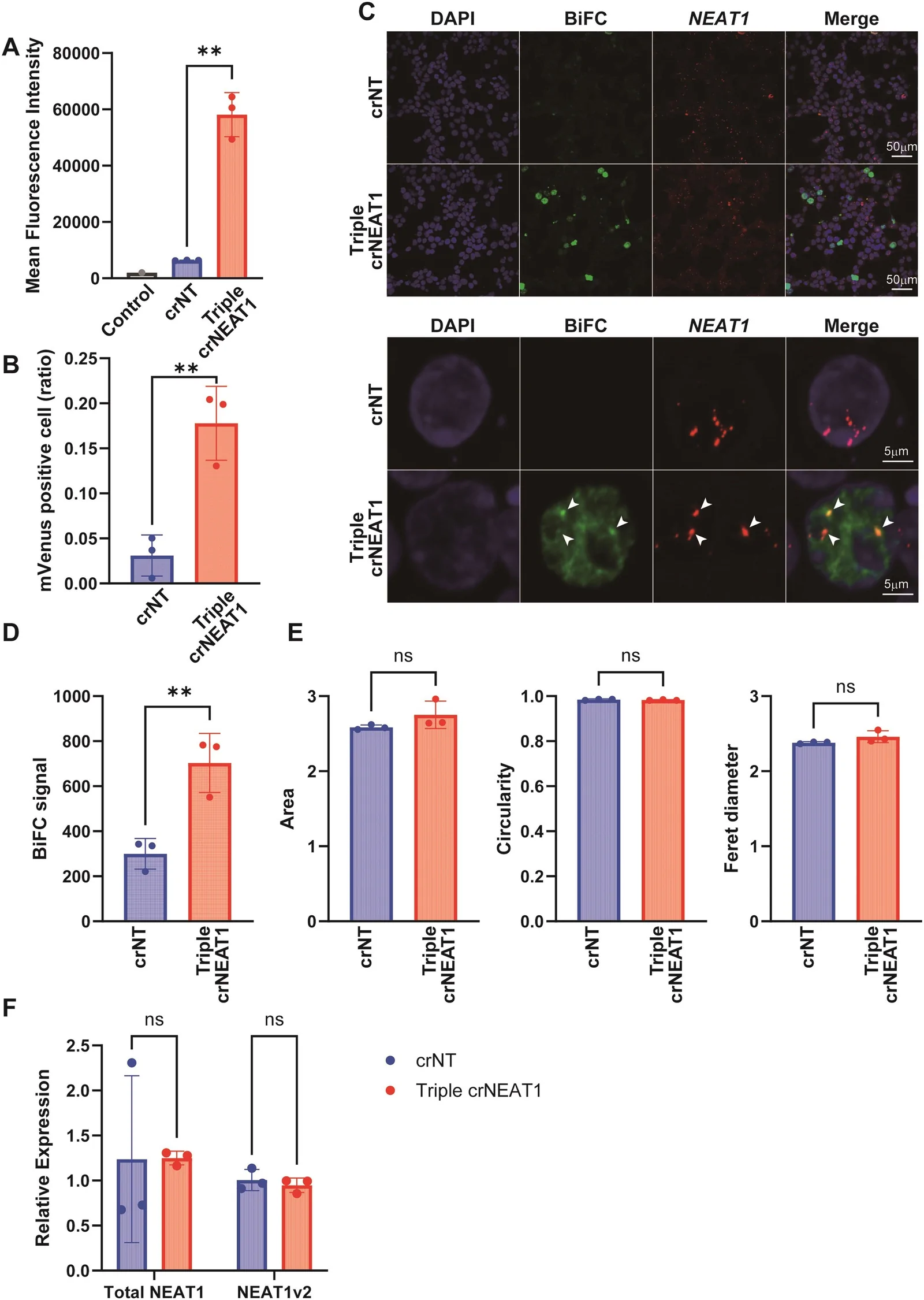
BiFC-dCas13 successfully records *NEAT1*–NONO interaction in paraspeckles without perturbing their integrity. (**A**) Quantification of mVenus fluorescence by flow cytometry in cells expressing the optimized BiFC-dCas13 system together with a control crRNA (crNT) or triple-cr*NEAT1* (a tandem crRNA construct containing cr*NEAT1*#2, #1, and #5). The mVenus fluorescence signal was measured 2 days after transfection. **P < .01, unpaired two-tailed *t*-test; *n* = 3 for each group. (**B**) Quantification of BiFC-positive cells in RNA FISH images. 293T cells expressing the optimized BiFC-dCas13 system together with crNT or triple-cr*NEAT1* were fixed 2 days after transfection and subjected to *NEAT1* RNA FISH. Cells were classified as BiFC-positive when the area occupied by the mVenus signal exceeded 10% of the DAPI-positive nuclear area. **P < .01, unpaired two-tailed *t*-test; *n* = 3 independent experiments per group. (**C**) Representative fluorescence images of 293T cells expressing the optimized BiFC-dCas13 system with crNT or triple-cr*NEAT1*. Cells were fixed 2 days after transfection and subjected to *NEAT1* RNA FISH. BiFC signals, *NEAT1* signals, and DAPI are shown. Upper panels show low-magnification views and lower panels show high-magnification views. Arrowheads indicate colocalization of BiFC and *NEAT1* signals. Scale bars: 50 μm for the upper panels and 5 μm for the lower panels. (**D**) Quantification of BiFC fluorescence intensity within *NEAT1*-positive regions identified by RNA FISH images. **P < .01, unpaired two-tailed *t*-test; *n* = 3 independent experiments per group. (**E**) Quantitative analysis of *NEAT1* foci morphology in cells expressing BiFC-dCas13 with crNT or triple-cr*NEAT1*. The area, circularity, and Feret diameter of *NEAT1*-positive foci are shown. Unpaired two-tailed *t*-test; *n* = 3 independent experiments per group. ns, not significant. (**F**) RT-qPCR analysis of total *NEAT1* and *NEAT1*v2 expression in cells expressing BiFC-dCas13 with crNT or triple-cr*NEAT1*. Data are presented as the mean ± SD. Statistical significance was determined using unpaired two-tailed *t*-test; *n* = 3 for each group. ns, not significant.

Collectively, these findings establish BiFC-dCas13 as an accumulative fluorescence reporter of *NEAT1*–NONO proximity, in which interaction events are recorded as persistent fluorescence signals that can be readily detected by microscopy and flow cytometry.

### BiFC-dCas13 minimally perturbs the native *NEAT1*–NONO complex

Because BiFC-dCas13 requires dCas13 binding to *NEAT1* together with the expression of the NONO-fusion component, we next examined whether the system itself perturbs the native *NEAT1*–NONO complex. Quantitative image analyses showed no significant differences in the number, size, or overall morphology of *NEAT1*-positive foci between crNT and cr*NEAT1* conditions (Fig. 3E and Supplementary Fig. S10C). Consistent with these observations, qPCR analysis revealed no significant changes in *NEAT1* expression (Fig. 3F). These results suggest that BiFC-dCas13 records *NEAT1*–NONO proximity without causing gross perturbation of endogenous *NEAT1* abundance or paraspeckle organization.

### Generation of the NanoBiT-dCas13 system

While the irreversible nature of BiFC complementation enables BiFC-dCas13 to record RPI events as accumulative fluorescence signals, it limits its ability to monitor dynamic changes in RPIs. To overcome this limitation and enable real-time monitoring of RPIs, we next developed a NanoBiT-based dCas13 platform. NanoBiT is a split luciferase consisting of LgBiT and SmBiT that reconstitutes functional NanoLuc only when these two molecules are in proximity to each other [3]. Importantly, the interaction between LgBiT and SmBiT is reversible, allowing this technology to be used to monitor dynamic protein association and dissociation.

We developed the NanoBiT-dCas13 system, in which the N- and C-terminal mVenus fragments were replaced by the LgBiT and SmBiT. In this system, the LgBiT and SmBiT interaction is induced by dCas13 targeting a specific RNA and a POI (Fig. 4A). Two combinations of the NanoBiT-dCas13 components (dCas13-LgBiT with NONO-SmBiT, and dCas13-SmBiT with NONO-LgBiT) were generated and transfected together with crRNAs in 293T cells. Combined expression of dCas13-LgBiT and NONO-SmBiT in conjunction with cr*NEAT1* resulted in the production of robust luminescence signals, whereas the combination of dCas13-SmBiT and NONO-LgBiT did not (Fig. 4B). Similar to BiFC-dCas13, the luciferase activity of NanoBiT-dCas13 with the multiplexed cr*NEAT1* was higher than that with a single cr*NEAT1* (Fig. 4B).

**Figure 4.**
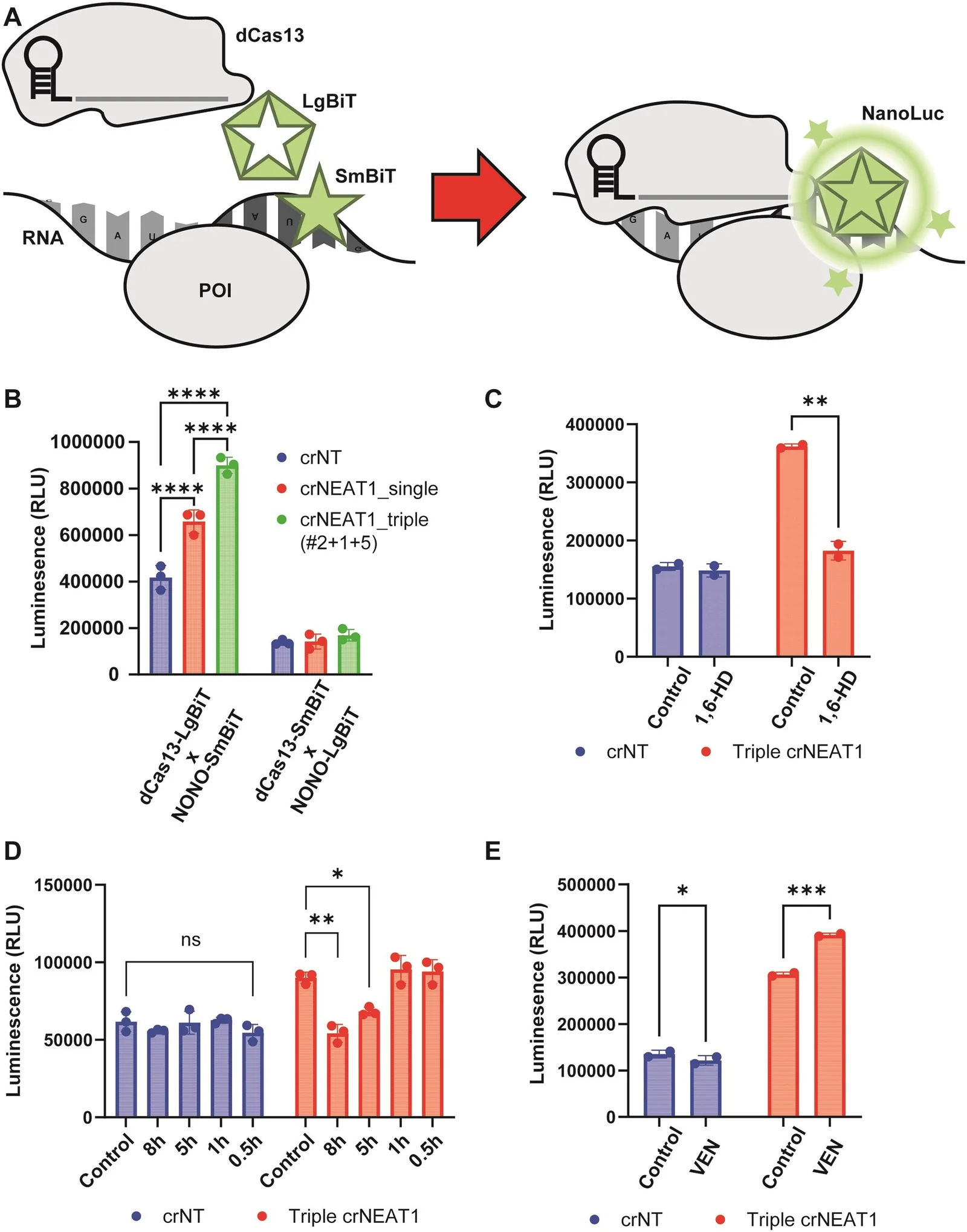
NanoBiT-dCas13 system detects paraspeckle formation in a reversible manner. (**A**) Schematic of the NanoBiT-dCas13 system. LgBiT and SmBiT are fused to dCas13d and a POI, respectively. dCas13d-LgBiT is directed to an ROI with a crRNA, and luciferase complementation is induced. When the complemented luciferase acts on the luciferin substrate, luminescence is emitted. (**B**) Luminescence intensity of different combinations of NanoBiT-dCas13 systems with crRNAs are shown as individual data points and the mean ± SD. 293T cells were transfected with NanoBiT-dCas13 components and crRNAs. Nano-Glo luciferin substrate was added, and luminescence was measured 2 days after transfection using a microplate reader. *n* = 3 for each group. ^****^P < .0001 (one-way ANOVA with Tukey–Kramer post hoc test). (**C**) Luminescence intensity of NanoBiT-dCas13d systems with crRNAs following 1,6-hexanediol (1,6-HD) treatment is shown as individual data points and the mean ± SD. 293T cells were transfected with NanoBiT-dCas13 components and crRNAs. Two days after the transfection, cells were treated with 5% 1,6-HD for 5 min and washed out. Nano-Glo luciferin substrate was added, and luminescence was measured using a microplate reader. *n* = 2 for each group. **P < .01 (two-way ANOVA with Sidak’s multiple comparisons test). (**D**) Luminescence intensity of NanoBiT-dCas13d systems with crRNAs following ActD treatment is shown as individual data points and the mean ± SD. 293T cells were transfected with NanoBiT-dCas13 components and crRNAs. Three days after the transfection, cells were treated with 1 μg/ml of ActD for indicated duration and washed out. Nano-Glo luciferin substrate was added, and luminescence was measured using a microplate reader. *n* = 3 for each group. *P < .05; **P < .01 (two-way ANOVA with Sidak’s multiple comparisons test). (**E**) Luminescence intensity of NanoBiT-dCas13d systems with crRNAs following venetoclax (VEN) treatment is shown as individual data points and the mean ± SD. 293T cells were transfected with NanoBiT-dCas13 components and crRNAs. Two days after the transfection, cells were treated with 10 μM of VEN for 30 min and washed out. Nano-Glo luciferin substrate was added, and luminescence was measured using a microplate reader. *n* = 2 for each group. *P < .05; ***P < .001 (two-way ANOVA with Sidak’s multiple comparisons test).

We investigated the reversibility of the NanoBiT-dCas13 system using 1,6-HD, a widely used reagent to dissolve LLPS-forming MLOs [23]. A 5-min incubation with 5% 1,6-HD completely abolished the luciferase activity of NanoBiT-dCas13 with cr*NEAT1* (Fig. 4C), demonstrating the reversibility of this system. We also monitored a time-dependent decrease in the *NEAT1*–NONO interaction signal following transcriptional inhibition with actinomycin D (ActD) (Fig. 4D). To investigate the stimuli that affect paraspeckle formation, we exposed 293T cells transfected with NanoBiT-dCas13 to various reagents. Interestingly, the BCL2 inhibitor venetoclax [24] enhanced the *NEAT1*–NONO interaction (Fig. 4E and Supplementary Fig. S12), suggesting the possible involvement of BCL2 in paraspeckle formation. Thus, we established the NanoBiT-dCas13 system to detect paraspeckle formation in a reversible manner.

### Generation of the split-TurboID-dCas13 system

Finally, we developed the split-TurboID-dCas13 system as a proximity-labeling platform for the discovery of molecules associated with specific RPI environments. In this system, N- and C-terminal fragments of TurboID, TbN73 and TbC74, are reconstituted in response to a specific RPI and biotinylate surrounding proteins in the presence of biotin (Fig. 5A). Because TurboID catalyzes proximity-dependent biotinylation, the system is expected to capture both stable and transiently associated factors within the local molecular environment. We constructed fusion proteins of dCas13 and NONO with N- or C-terminal TurboID, generating several dCas13-split TurboID fusions (dCas13-TbN73_v2 or dCas13-TbC74_v2) and NONO-split TurboID fusions (NONO-TbN73/TbC74; TbN73/TbC74-NONO) (Supplementary Fig. S13). Co-transfection of dCas13-TbN73_v2, NONO-TbC74, and cr*NEAT1* into 293T cells induced protein biotinylation, and the biotinylated proteins were enriched by SA bead affinity purification (Fig. 5B). We then evaluated the biotinylation-inducing activity of four sets of the split-TurboID-dCas13 system co-expressed with single, double (cr*NEAT1*#2 + 1), or triple (cr*NEAT1*#2 + 1 + 5) cr*NEAT1*, and found that dCas13-TbN73_v2 and NONO-TbC74 together with triple cr*NEAT1* was the best combination to efficiently induce biotinylation of nearby proteins (Fig. 5C).

**Figure 5.**
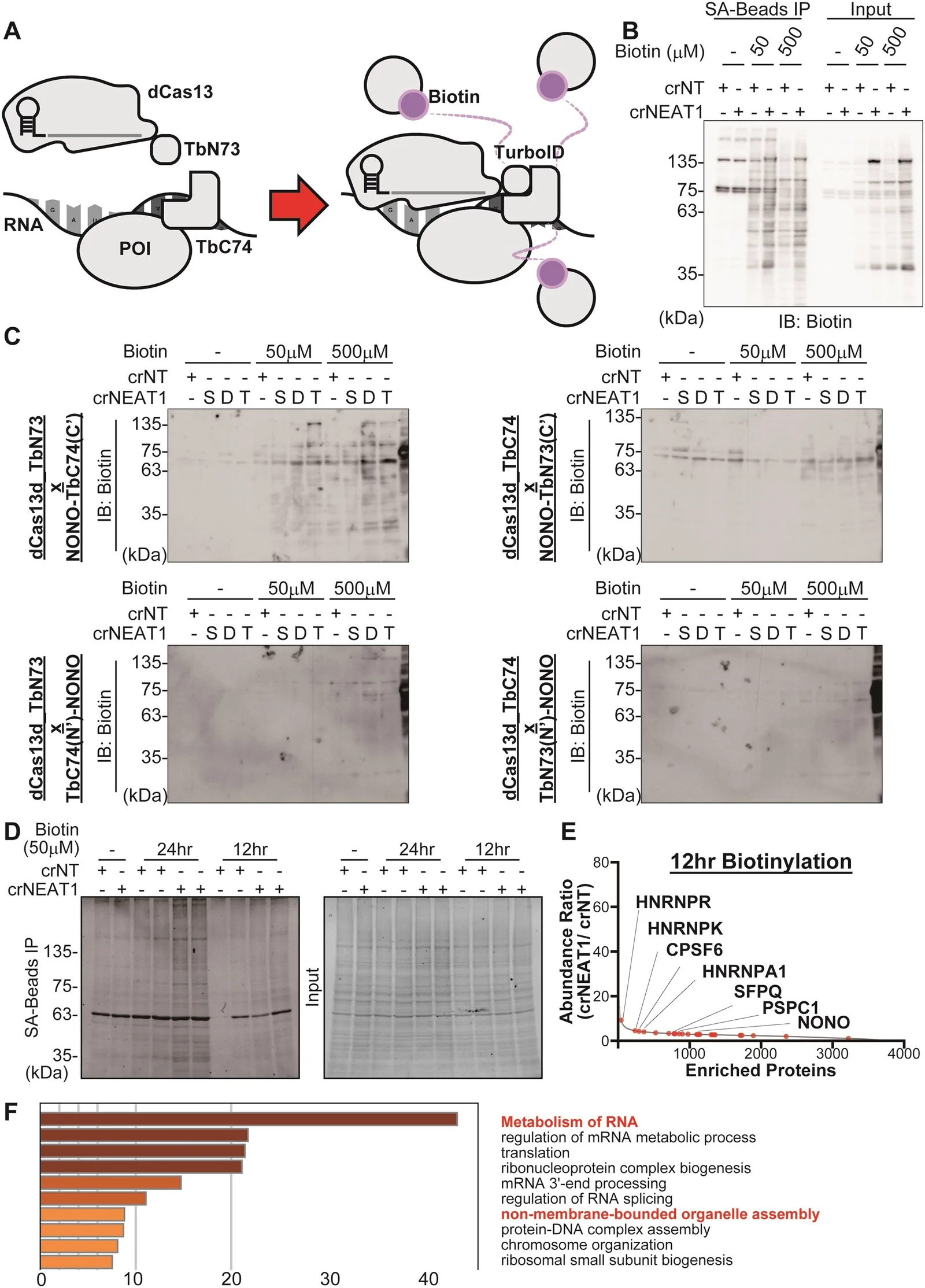
Split-TurboID dCas13 system enables proximity labeling of proteins surrounding paraspeckles. (**A**) Schematic of the split-TurboID-dCas13 system. TbN73 and TbC74 are fused to dCas13d and a POI, respectively. dCas13d-TbN73 is directed to an ROI with a crRNA, and BL complementation is induced. In the presence of biotin, BL biotinylates proteins surrounding the POI–ROI complex. (**B**) Representative biotin immunoblot of SA affinity purification from 293T cells transfected with the split-TurboID-dCas13 system. 293T cells were transfected with dCas13d-TbN73_v2, NONO-TbC74, and crRNAs. Two days after transfection, biotinylation was induced at the indicated concentration for 24 h. Total cell lysates were isolated and purified with SA beads, and biotinylated proteins were detected with an anti-biotin antibody. (C) Representative biotin immunoblot of four different combinations of split-TurboID-dCas13 systems. 293T cells were transfected with crRNA and split-TurboID-dCas13 components in different combinations as follows: dCas13d-TbN73_v2 and NONO-TbC74 (NONO-TbC), dCas13d-TbC74_v2 and NONO-TbN73 (NONO-TbN), dCas13d-TbN73_v2 and TbC74-NONO (TbC-NONO), and dCas13d-TbC74_v2 and TbN73-NONO (TbN-NONO). Two days after transfection, biotinylation was induced at the indicated concentration for 24 h. Total cell lysates were subjected to western blotting with an anti-biotin antibody. S, single-cr*NEAT1*; D, dual-cr*NEAT1*; T, triple-cr*NEAT1*. (**D**) Oriole staining after SA affinity purification of 293T cells transfected with optimized split-TurboID-dCas13 systems. 293T cells were transfected with dCas13d-TbN73_v2, NONO-TbC74, and crNT or triple-cr*NEAT1*. Two days after transfection, cells were incubated with 50 μM biotin for the indicated time course. Total cell lysates were isolated and purified with SA beads. Enriched proteins were analyzed by Oriole staining. (**E**) Fold change (cr*NEAT1*/crNT) in protein abundance. Affinity-purified proteins after 12 h of biotinylation induced by the split-TurboID-dCas13 systems were subjected to LC-MS/MS analysis. Red dots indicate proteins known as paraspeckle components [25]. *n* = 2 for each group. See also Supplementary Fig. S14B. (**F**) Enriched ontology clusters of the split-TurboID-dCas13 with cr*NEAT1*. Enriched proteins in the cr*NEAT1* group (FDR < 0.2 in both biotinylations) were analyzed by Metascape [26].

Using this split-TurboID-dCas13 system, we next performed an interactome analysis around the *NEAT1*–NONO complex in paraspeckles. We transfected constructs of the split-TurboID-dCas13 system into 293T cells and induced biotinylation for 12 or 24 h. Biotinylated proteins were then collected using SA beads and subjected to liquid chromatography-tandem mass spectrometry (LC-MS/MS) (Fig. 5D). Similar proteins were biotinylated at both 12 and 24 h (Supplementary Fig. S14A). LC-MS/MS analysis revealed that known paraspeckle proteins, such as SFPQ and PSPC1 [25], were biotinylated in cr*NEAT1*-transfected 293T cells (Fig. 5E and Supplementary Fig. S14B). Enrichment analysis using Metascape [26] showed that proteins related to RNA metabolism and those that assemble non-membrane-bound organelles were highly enriched (Fig. 5F). These results demonstrate that the *NEAT1*–NONO-targeting split-TurboID-dCas13 system can enrich proteins associated with the molecular environment surrounding the *NEAT1*–NONO interaction, including known paraspeckle components. Given the cumulative nature of the 12- and 24-h biotinylation, the identified proteins likely include proteins encountered by the *NEAT1*–NONO-targeted split-TurboID complex both within and outside paraspeckles, rather than representing a spatially restricted paraspeckle proteome.

### YTHDC1, an m^6^A reader protein, supports paraspeckle formation

Among the proteins enriched by the split-TurboID-dCas13 system, m^6^A RNA regulatory proteins, particularly m^6^A “reader” proteins, were prominent (Fig. 6A). Given the cumulative nature of the proximity-labeling approach described earlier, we next sought to independently examine the biological relevance of one of these candidates. We focused on YTHDC1 because it localizes to the nucleus where paraspeckles form. We first sought to validate the relationship between YTHDC1 and the *NEAT1*–NONO molecular environment. RIP analysis demonstrated robust enrichment of *NEAT1* by YTHDC1 (Fig. 6B and Supplementary Fig. S15A). In contrast, co-immunoprecipitation analysis failed to detect a stable interaction between YTHDC1 and NONO (Supplementary Fig. S15B). These results suggest that YTHDC1 is associated with *NEAT1* despite the absence of a detectable stable interaction with NONO.

**Figure 6.**
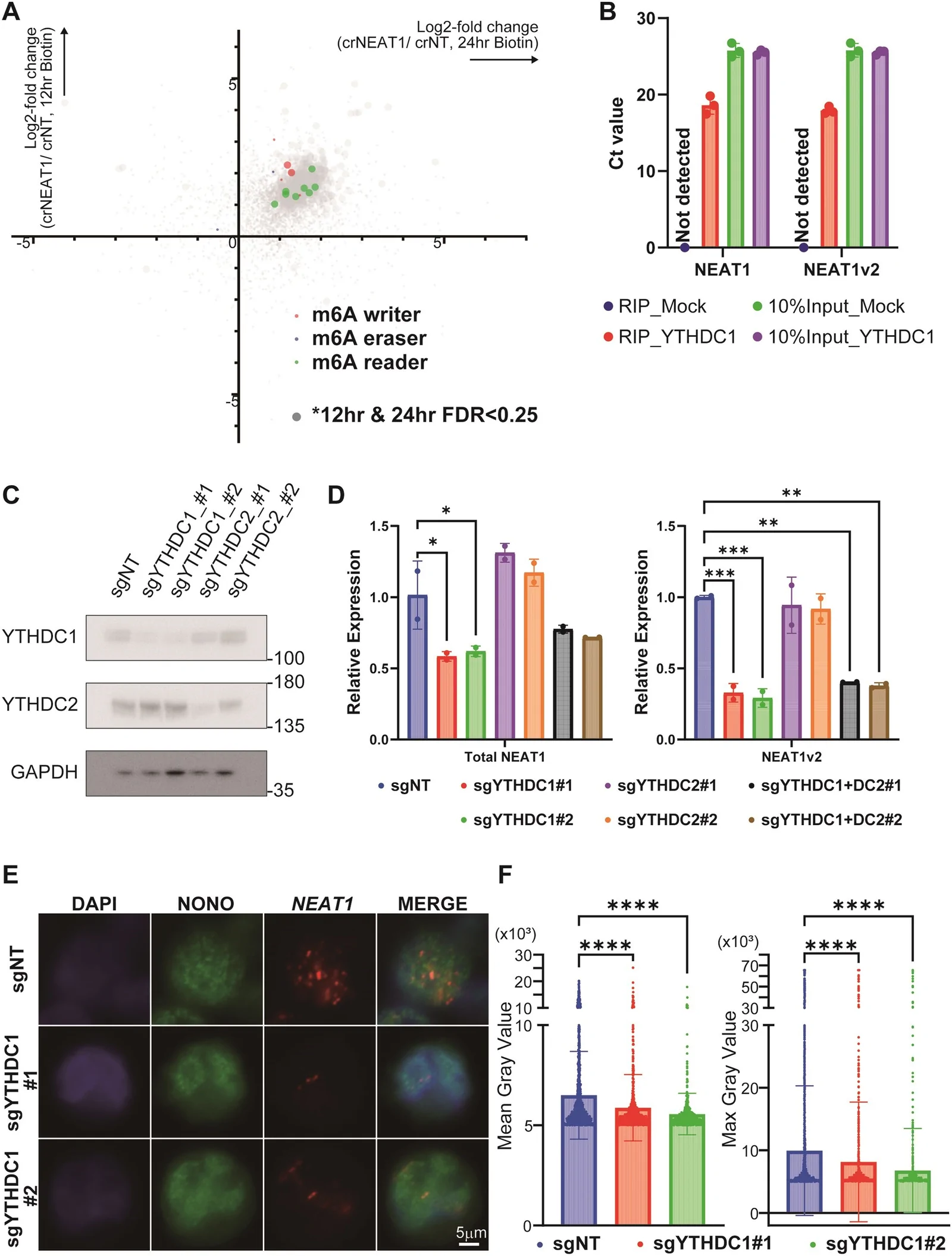
YTHDC1 supports paraspeckle formation through *NEAT1* stabilization. (**A**) Scatter plot showing the log_2_-fold change (cr*NEAT1*/ crNT, 24-h biotinylation) versus the log_2_-fold change (cr*NEAT1*/crNT, 12-h biotinylation) for each protein related to Fig. 5F. Red, blue, and green dots indicate m^6^A RNA regulatory proteins: writers, erasers, and readers, respectively. Large dots indicate proteins that are significantly enriched by cr*NEAT1* (FDR < 0.25) both at 12 and 24 h. (**B**) Enrichment of *NEAT1* following FLAG-YTHDC1 RIP. 293T cells were transfected with a mock vector or FLAG-YTHDC1. Nuclear lysates were subjected to anti-FLAG immunoprecipitation, and co-immunoprecipitated RNAs were analyzed by RT-qPCR. Ct values for *NEAT1* and *NEAT1* variant 2 (*NEAT1*v2) are shown. Input samples (10% input) are presented as controls. *n* = 3 independent experiments. (**C**) Depletion of YTHDC1 and YTHDC2 in K562 cells was confirmed by western blotting. Cells were transduced with non-targeting (NT), YTHDC1-targeting (sgYTHDC1), or YTHDC2-targeting sgRNA (sgYTHDC2) (co-expressing a puromycin resistance gene). Total protein was extracted from cells after 3 days of puromycin selection. (**D**) Relative expression of total *NEAT1* and *NEAT1*v2 in YTHDC1- or YTHDC2-depleted K562 cells is shown as the means ± SDs. K562 cells expressing Cas9 were transduced with sgNT, sgYTHDC1, or sgYTHDC2 (co-expressing a puromycin resistance gene). RNA was extracted from cells after puromycin selection and subjected to RT-qPCR. Data were analyzed after TBP normalization. *P < .05; **P < .01; ***P < .001 (one-way ANOVA with Dunnett post hoc test). *n* = 2 (technical replicates) for each group. (**E**) RNA FISH and immunofluorescence images of YTHDC1-depleted K562 cells. K562 cells expressing Cas9 were transduced with sgNT or sgYTHDC1 (co-expressing a puromycin resistance gene). Cells were fixed 2 days after puromycin selection. Fluorescence images were acquired using the Nikon A-1 microscope imaging system. *NEAT1*v2, NONO, and DAPI-stained nuclei are shown. Representative images are shown. Scale bars: 5 μm. (**F**) Mean (left) and maximum (right) *NEAT1* signal intensities in each cell are shown as individual data points and the mean ± SDs. Signal intensities were measured from 2773, 1082, and 779 cells, respectively. ^****^P < .0001 (one-way ANOVA with Dunnett post hoc test).

To further characterize the spatial relationship between YTHDC1 and paraspeckles, we established a reporter cell line to visualize endogenous YTHDC1 together with the paraspeckle protein NONO using mCherry and mNeonGreen, respectively (Supplementary Fig. S16A). We first introduced the sequence of the small mNeonGreen fragment (mNG_2_11) [27] into the C-terminal locus of the *NONO* gene to express the NONO-mNG_2_11 fusion protein in K562 cells expressing the large mNeonGreen fragment [mNG_2_(1–10)]. In this reporter cell line, the NONO-mNG_2_11 complements with mNG_2_(1–10) to restore fluorescence reflecting NONO expression. We also introduced the mCherry sequence into the N-terminus locus of the *YTHDC1* gene in K562 cells and established an mCherry-YTHDC1 reporter cell line and a NONO/YTHDC1 dual-reporter cell line (Supplementary Fig. S16B and C). Successful knock-in in these reporter cell lines was confirmed by PCR (Supplementary Fig. S17). Live-cell imaging of the dual- reporter cells revealed the broad coexistence of NONO and YTHDC1 (Supplementary Figs S16C and S18), indicating a close association between YTHDC1 and paraspeckles. However, compared to the signal of NONO, which forms paraspeckle foci in the nucleus, the YTHDC1 signal was flatter, more diffuse and localized “around” the peak of the NONO signal (Supplementary Fig. S18). Therefore, YTHDC1 is unlikely to be a core component of paraspeckles, but rather a cooperating protein localized to the periphery of paraspeckles.

Since m^6^A reader proteins regulate the fate of RNAs, we speculated that m^6^A readers are associated with paraspeckle formation through *NEAT1* regulation. To test this possibility, we depleted YTHDC1 and its paralog, YTHDC2, using the CRISPR/Cas9 system in K562 cells (Fig. 6C). Depletion of YTHDC1, but not YTHDC2, induced a significant reduction in *NEAT1* expression (Fig. 6D). NONO immunofluorescence coupled with *NEAT1* RNA FISH also revealed a reduction of paraspeckles in YTHDC1-depleted K562 cells (Fig. 6E and F). Taken together, these results suggest that YTHDC1 coexists with NONO, although not necessarily within the paraspeckle foci, and functionally supports paraspeckle formation by stabilizing *NEAT1*.

### Adaptation of BiFC-dCas13 to distinct RPI pairs

Finally, we examined whether the dCas13-based RPI analysis platforms could be extended beyond the original *NEAT1*–NONO paraspeckle model. Using BiFC-dCas13 as a representative platform and YTHDC1 as an alternative protein partner, we redesigned either the protein fusion component or the RNA-targeting crRNA to test the modularity of the system.

We first tested protein-component flexibility by generating YTHDC1-BiFC fusion proteins through the fusion of either VN155 or VC155 to the N-terminus of YTHDC1 (Fig. 7A). These constructs were co-expressed with BiFC-dCas13 and the optimized *NEAT1*-targeting crRNA combination (triple cr*NEAT1*#2 + 1 + 5) in 293T cells. Flow cytometric analysis revealed robust mVenus signals in cells expressing cr*NEAT1* compared with non-targeting controls (Fig. 7B), demonstrating the successful adaptation of the BiFC-dCas13 platform to the YTHDC1–*NEAT1* interaction pair. These results indicate that the interaction framework identified by split-TurboID-dCas13 can be successfully reconstituted using the BiFC-dCas13 platform.

**Figure 7.**
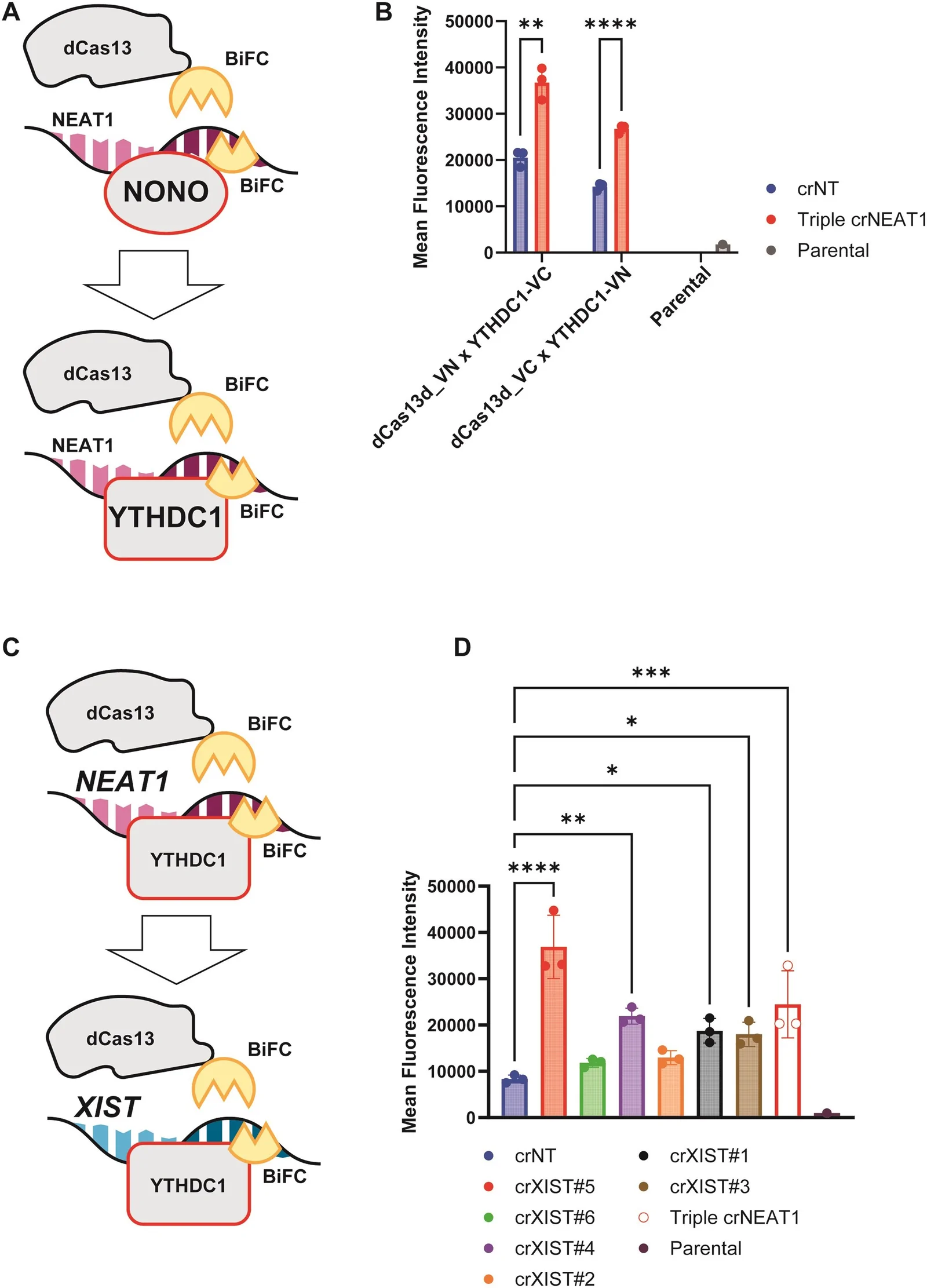
Adaptation of BiFC-dCas13 to distinct RPI pairs. (**A**) Schematic illustration of the BiFC-dCas13 system reconfigured from the *NEAT1*–NONO interaction pair to the *NEAT1*–YTHDC1 interaction pair. (**B**) Quantification of mVenus fluorescence following the replacement of NONO with YTHDC1 as the protein fusion partner. 293T cells were transfected with BiFC-dCas13, YTHDC1-BiFC fusion constructs, and either crNT or triple-cr*NEAT1* (a tandem crRNA construct containing cr*NEAT1*#2, #1, and #5). Mean fluorescence intensity was measured by flow cytometry 2 days after transfection. **P < .01, ^****^P < .0001, unpaired two-tailed *t*-test; *n* = 3 for each group. (**C**) Schematic illustration of the BiFC-dCas13 reconfigured from the YTHDC1–*NEAT1* interaction pair to the YTHDC1–*XIST* interaction pair. (**D**) Quantification of mVenus fluorescence following the replacement of the RNA target from *NEAT1* to *XIST*. 293T cells were transfected with BiFC-dCas13, YTHDC1-BiFC fusion constructs, and the indicated *XIST*-targeting crRNAs. Mean fluorescence intensity was measured by flow cytometry 2 days after transfection. *P < .05, **P < .01, ***P < .001, ^****^P < .0001, one-way ANOVA with Dunnett post hoc test; *n* = 3 for each group.

YTHDC1 is a well-characterized m^6^A reader that associates with multiple transcripts, including the long non-coding RNA *XIST*, a key mediator of X-chromosome inactivation [9]. We therefore redirected the BiFC-dCas13 system from the YTHDC1-*NEAT1* interaction pair to YTHDC1–*XIST* in order to examine the RNA-component flexibility of the platform (Fig. 7C). *XIST*-targeting crRNAs were designed (Supplementary Fig. S19) and tested for their ability to induce BiFC signals in combination with VC155-YTHDC1 and dCas13-VN155. Flow cytometric analysis showed that multiple *XIST*-targeting crRNAs induced robust BiFC signals (Fig. 7D). These results demonstrate the successful adaptation of the BiFC-dCas13 platform to the YTHDC1–*XIST* interaction pair.

Together, these results demonstrate that BiFC-dCas13 can be readily reconfigured to detect distinct RPI pairs through the simple redesign of either the RNA-targeting guide or the protein fusion component.

## Discussion

In this work, we developed three complementary CRISPR/Cas13-based platforms to record, monitor, and discover RPI-associated biology (Supplementary Fig. S20). Among them, BiFC-dCas13 functions as an accumulative fluorescence recorder in which RPI events are integrated into persistent fluorescence signals through irreversible fluorophore complementation. As proof-of-concept applications, BiFC-dCas13 successfully detected the *NEAT1*–NONO, *NEAT1*–YTHDC1, and *XIST*–YTHDC1 interaction pairs. Unlike conventional dCas13-based imaging approaches [5], fluorescence generation in BiFC-dCas13 is directly coupled to RNA–protein proximity rather than simple RNA recognition. Consequently, fluorescence intensity reflects the cumulative history of interaction events, enabling quantitative readouts by flow cytometry, microscopy, or plate-reader-based analysis. Another technology used for RNA tracking is the MS2-MCP system, in which RNAs tagged with MS2 sequences interact with MCP [28]. Because MS2 sequences must be present in the target transcript, this system is not ideally suitable for tracking endogenous RNAs. Combined with the ease of crRNA design, BiFC-dCas13 provides a versatile approach for recording endogenous RPIs without the need for genetic engineering of the target transcript.

The irreversible nature of BiFC complementation represents both a strength and a limitation of the platform. On one hand, irreversible fluorophore formation enables the accumulation and recording of RPI events over time, thereby providing a highly sensitive fluorescence-based readout. On the other hand, once formed, the fluorophore remains fluorescent even after the dissociation or relocation of the original interaction partners. Consequently, BiFC-dCas13 is not ideally suited for strict real-time spatial interpretation of RPIs. We have therefore developed the NanoBiT-dCas13 system, which provides a reversible luminescence readout that is better suited for monitoring dynamic changes in RPIs. NanoBiT-dCas13 can quantify the strength and dynamics of RPIs using a plate-reader-based analysis, and will be particularly suitable for high-throughput screening using CRISPR-based gRNA libraries and/or compound libraries, which may lead to a paradigm shift in the discovery of new drugs targeting endogenous RPIs in the future.

MLOs are composed of numerous molecules and play important roles in diverse biological processes. Our split-TurboID-dCas13 system is highly effective for discovering proteins associated with specific RPI environments across diverse types of MLOs. In contrast to BiFC-dCas13 and NanoBiT-dCas13, which record and monitor RPIs, respectively, split-TurboID-dCas13 serves as a discovery platform for uncovering molecular components associated with specific RPI environments. Several RNA-centered proximity-labeling approaches have previously been developed to identify molecules associated with specific transcripts, including systems that combine programmable RNA-targeting modules with proximity-labeling enzymes [6, 29]. Our split-TurboID-dCas13 platform was inspired by these approaches and shares the common goal of interrogating RNA-associated molecular environments. By leveraging split-TurboID-mediated proximity labeling, we extended this concept from transcript-centered proximity labeling to RPI-centered molecular discovery. Consequently, the system enables the interrogation of molecular environments surrounding specific RPI frameworks, providing a complementary perspective to existing transcript-centered proximity-labeling approaches. As a proof of concept, we applied split-TurboID-dCas13 to investigate the molecular environment surrounding the *NEAT1*–NONO interaction framework and identified YTHDC1 as a candidate factor associated with this environment. The association between YTHDC1 and *NEAT1* has been reported previously [30], and a recent study showed that YTHDC1 itself forms condensates to suppress myeloid differentiation [31]. Our findings further place YTHDC1 within the *NEAT1*–NONO-centered molecular environment captured by split-TurboID-dCas13. Together with RIP and functional analyses, these results support the utility of split-TurboID-dCas13 as a discovery platform for identifying biologically relevant components associated with specific RPI networks. Since proximity-labeling technologies are now used to capture nucleic acids [32], the split-TurboID-dCas13 system can also be applied to identify DNAs and RNAs that interact with specific MLOs. Indeed, paraspeckles and *NEAT1* have been shown to interact with multiple RNAs and genomic loci [33, 34], which can be systematically identified using the split-TurboID-dCas13 system.

Because our platforms rely on the exogenous expression of dCas13-fusion constructs, the possibility of perturbing native RPI networks should be considered. We therefore view the outputs of these systems as starting points for biological investigation rather than definitive evidence of physiological interactions. The identification and subsequent validation of YTHDC1 through orthogonal approaches illustrates the importance of integrating complementary experimental strategies when interpreting results obtained from engineered RPI-analysis platforms. The effective spatial and temporal resolution of split-TurboID-dCas13 also remains to be defined. Because the 12- and 24-h labeling profiles obtained in this study reflect cumulative proximity labeling rather than instantaneous interactions, future time-course and distance-calibration studies will be required to determine the labeling range of the platform.

The present study focused on Cas13d because of its compact size and robust target specificity. However, a growing number of RNA-targeting CRISPR effectors, including smaller Cas13 orthologs such as Cas13X [35], have recently been described. Future studies should investigate whether alternative Cas13 proteins further improve the performance of these platforms, as differences in protein size, structure, and fusion-partner tolerance may influence signal generation, labeling efficiency, and adaptability to diverse RPI systems. In addition, the Cas13-based RPI analytical tools developed in this study contain multiple NLS sequences and mainly target nuclear RNAs and proteins. However, there are numerous cytoplasmic MLOs, including stress granules and P-bodies whose functions are not fully understood [36]. The development of similar tools targeting cytoplasmic MLOs is an important future challenge. Moreover, the incorporation of diverse split effectors, including reversible fluorescence complementation systems, may further expand the toolkit for monitoring RPI dynamics in living cells.

To study endogenous RPIs under physiological conditions, it will also be important to combine the knock-in technology with our systems. For example, we can introduce split mVenus or split TurboID fragment sequences into the N- or C-terminus of the POI (e.g. NONO). In parallel, targeted integration of dCas13-fusion constructs into safe-harbor loci may help achieve more controlled expression levels and reduce potential artifacts associated with transient overexpression. Although BiFC-dCas13 did not grossly alter *NEAT1* abundance or paraspeckle morphology, subtle effects of the fusion constructs on RNP assembly, exchange kinetics, or molecular residence time cannot be excluded. Endogenous tagging and expression-level-controlled systems will therefore be important for evaluating RPI dynamics under more physiological conditions.

In summary, we combined RNA-targeting dCas13 with split-protein technologies to establish a suite of complementary platforms for recording, monitoring, and discovering RPI-associated biology. We further demonstrated the modularity of this framework by extending it from the original *NEAT1*–NONO interaction pair to additional RPI systems. Together, these tools provide a versatile foundation for the detection, analysis, and discovery of endogenous RPIs and their associated molecular environments.

## Supplementary Material

gkag928_Supplemental_Files

## Data Availability

The mass spectrometry proteomics data have been deposited to the ProteomeXchange Consortium via the PRIDE partner repository with the dataset identifier PXD066895. The original western blot and gel electrophoresis data are available on Mendeley Data: doi: 10.17632/67dv9cnxjt.1. Plasmids generated in this study are currently being deposited with Addgene and have been assigned plasmid IDs 260608 and 262569–262576. They will be made publicly available upon completion of the deposition process. In the interim, plasmids are available from the corresponding author upon reasonable request.
