# Supplementary material for: Development of CRISPR/Cas13-based analytical tools to study RNA–protein interactions
Source: Nucleic Acids Res. 2026 Oct 13;54(18):gkag928. doi: 10.1093/nar/gkag928 (PMC13657679; doi:10.1093/nar/gkag928)
Supplement: Supplementary file 1 [file gkag928_supplemental_files.zip › Supplementary File_NAR_v4_Re-Revision_260901.pdf]

Supplementary Figure and Supplementary Figure Legends

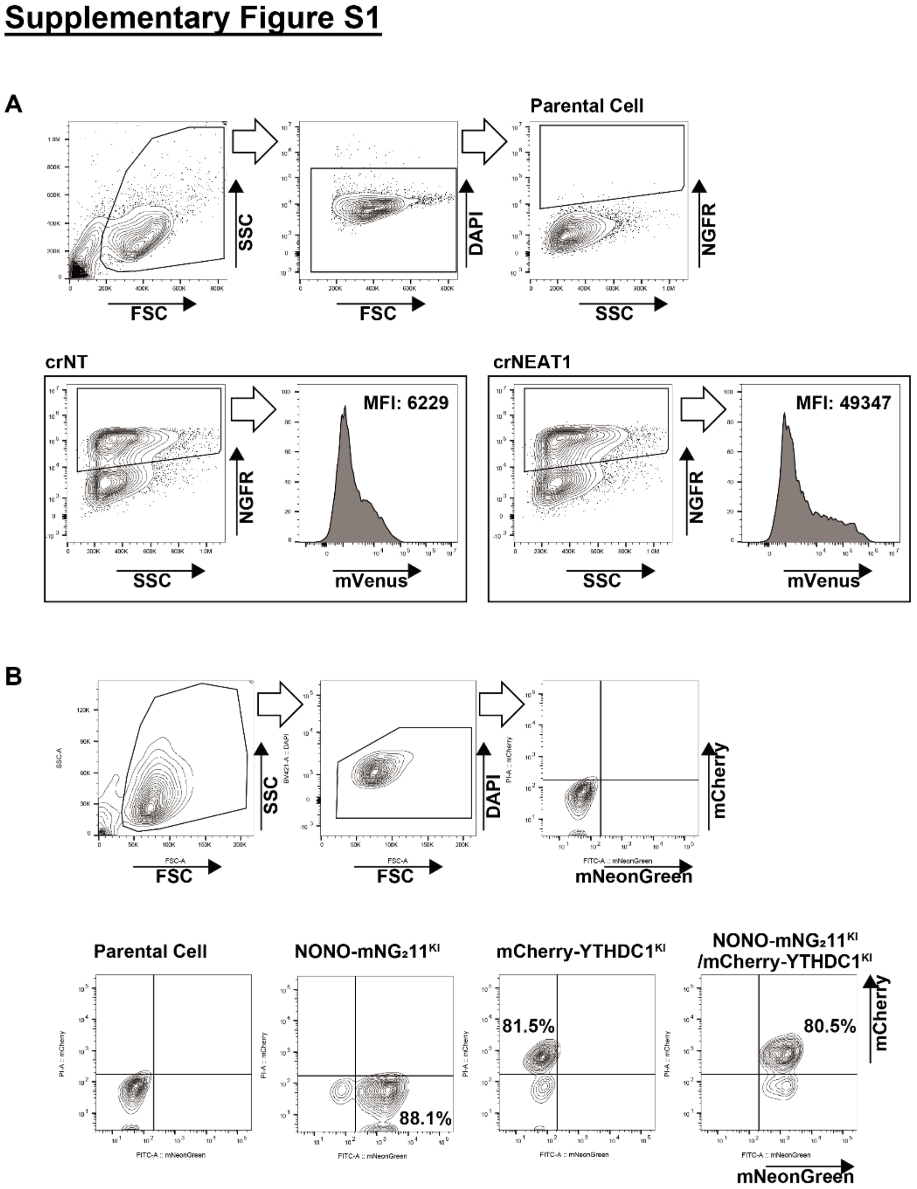

Supplementary Figure S1. Schematic of flow cytometry analysis

A. Schematic of the NGFR-based gating strategy used to analyze 293T cells transiently transfected with the BiFC-dCas13 system is shown (top). Representative flow cytometry plots for the crNT and cr*NEAT1* conditions are shown (bottom).

B. Schematic of flow cytometry analysis of knock-in K562 cells is shown (top). Representative flow cytometry plots of parental, NONO-mNG$_2$11-KI, mCherry-YTHDC1-KI, and double knock-in cells are shown (bottom).

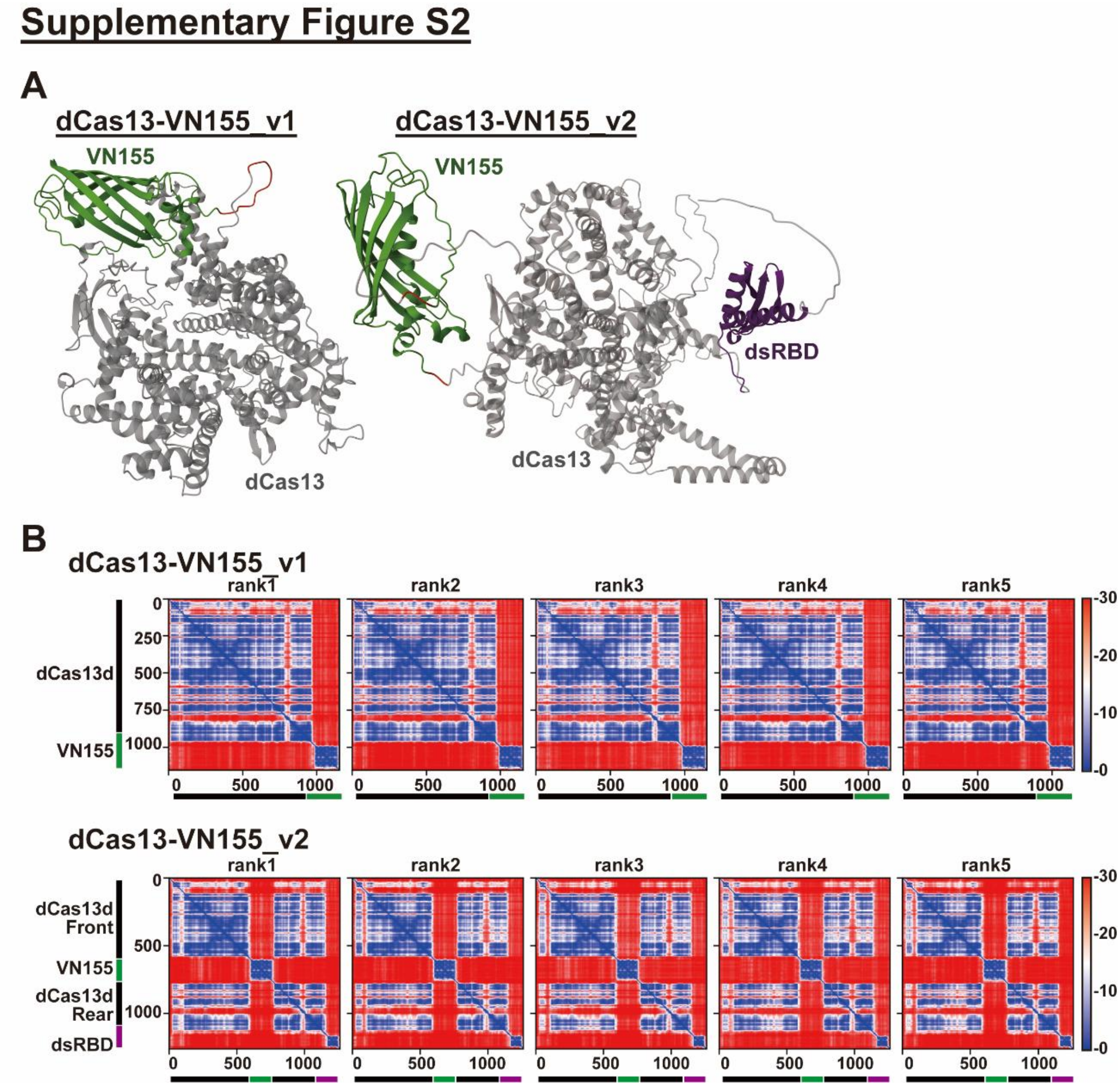

Supplementary Figure S2 (related to Figure 1). Prediction models of the BiFC-dCas13d protein structure.

A. 3D protein structures of BiFC-dCas13d_v1 and BiFC-dCas13d_v2 predicted by AlphaFold. Regions colored in gray, red, green, and purple indicate dCas13d, the flexible linker, VN155, and the dsRBD, respectively.

B. Predicted aligned error maps of dCas13d-VN155_v1 (upper panels) and dCas13d-VN155_v2 (lower panels) was predicted from amino acid sequences by AlphaFold. Five different models and their predictions are shown.

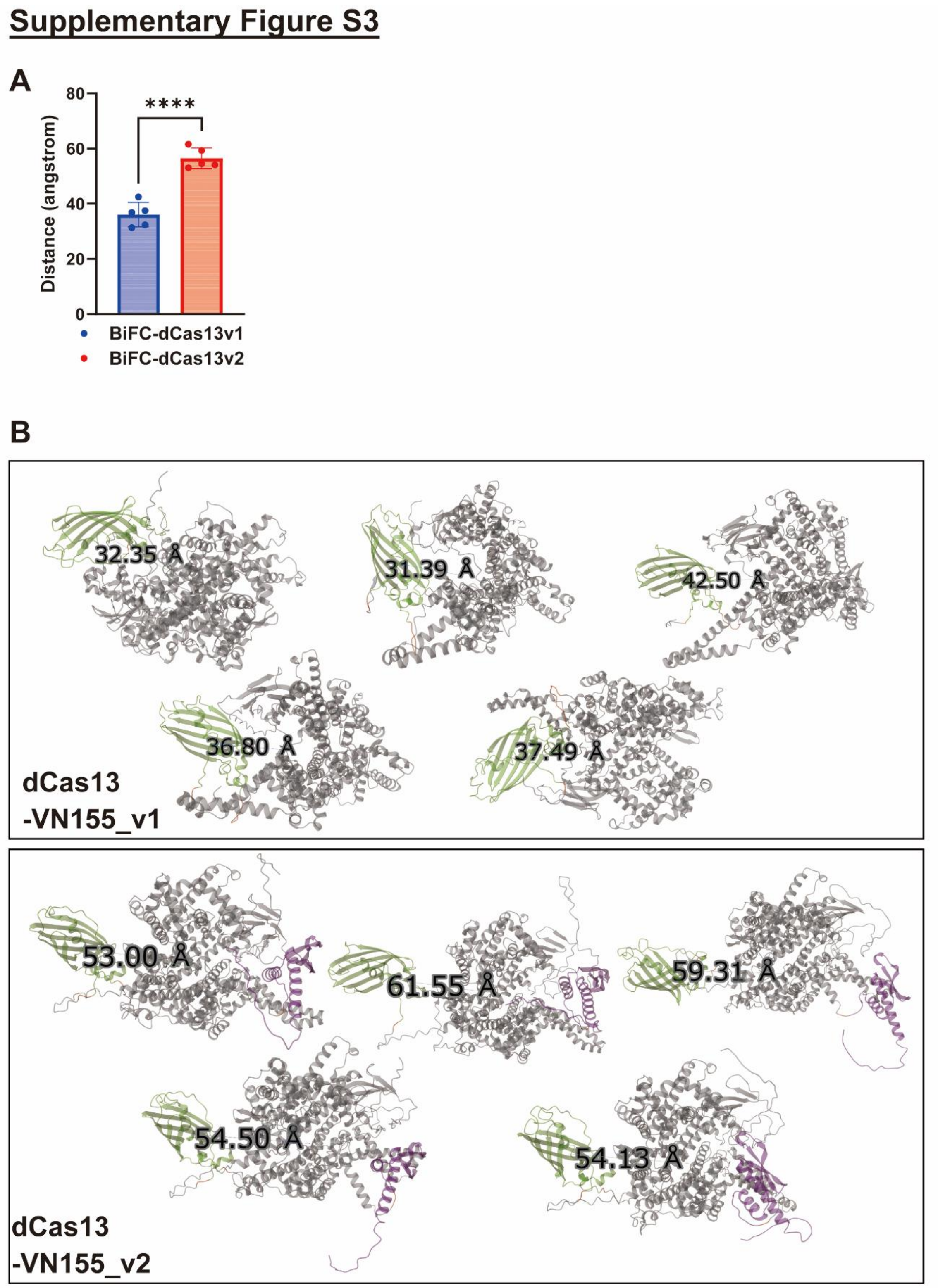

Supplementary Figure S3 (related to Figure 1). The molecular distance between dCas13d and VN155 in prediction models.

A. The molecular distances between dCas13d and VN155 in the prediction models of version 1

and version 2 are shown as individual data points and the mean +- SDs. For each of the dCas13d-VN155 version 1 and version 2 constructs, five prediction models were obtained from AlphaFold. Based on these models, the center positions of dCas13d and VN155 were determined and the distance between these center positions was calculated using MolStar analysis. ****$P < 0.0001$ (unpaired t test). n = 5 for each group.

B. The five different prediction models of the BiFC-dCas13d_v1 and BiFC-dCas13d_v2 structures used in the calculation for Supplementary Figure S3A are shown. Molecular distances between dCas13d and VN155 were calculated using MolStar (https://molstar.org/). The distance between the center positions of dCas13d and VN155 calculated from each of the models is also shown.

Supplementary Figure S4

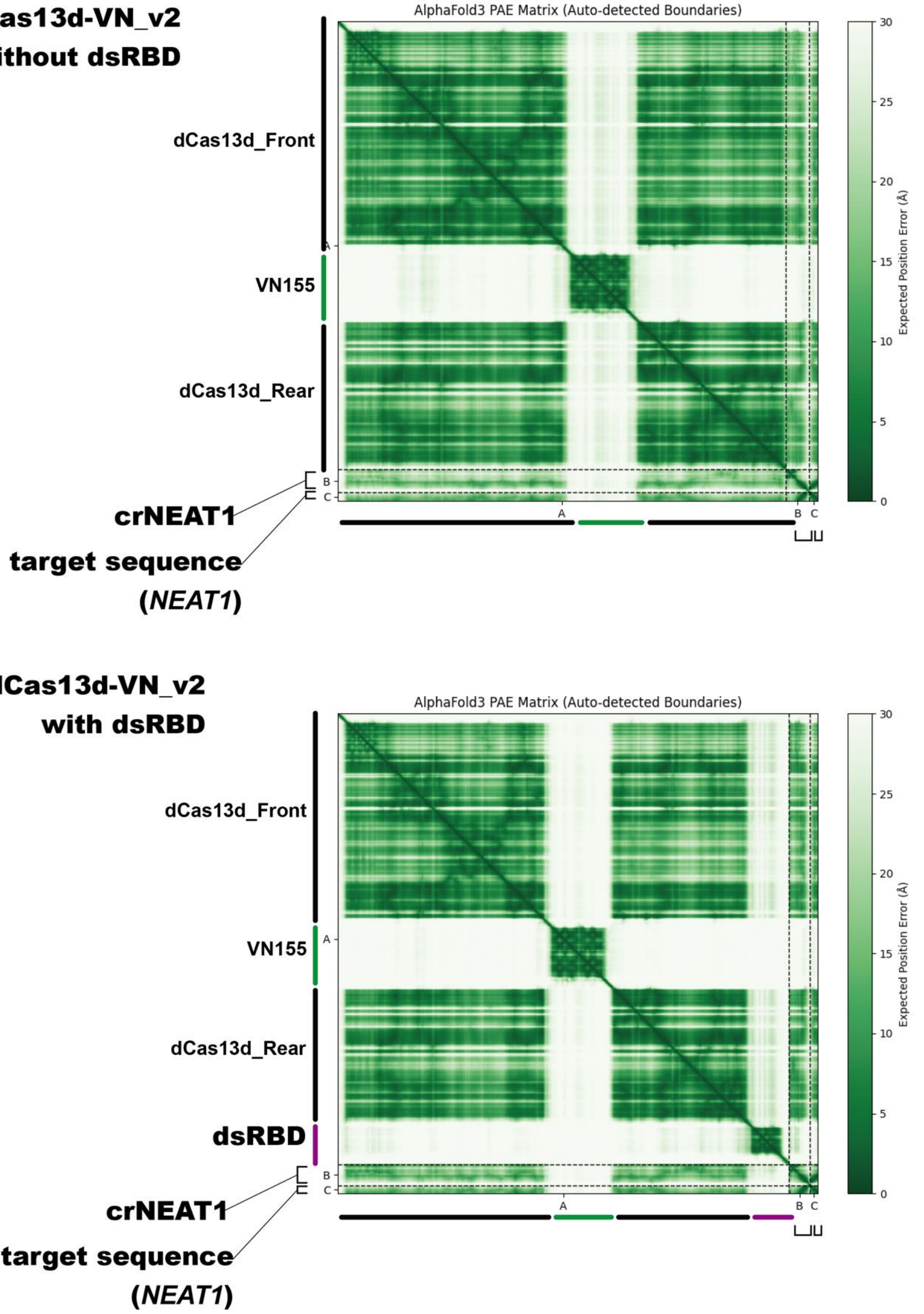

Supplementary Figure S4 (related to Figure 1). Predicted aligned error matrices of the BiFC-dCas13d–crRNA–target RNA complexes with or without the appended dsRBD.

Predicted aligned error (PAE) matrices for AlphaFold3 models of the BiFC-dCas13d_v2–cr*NEAT1*–target RNA complex without the dsRBD (upper) and with the appended dsRBD (lower) are shown. The boundaries corresponding to the dCas13d N-terminal region, VN155, dCas13d C-terminal region, dsRBD, cr*NEAT1*, and the *NEAT1* target RNA sequence are indicated. PAE values are displayed on a scale from 0 to 30 Å, with lower values indicating greater confidence in the relative positioning of the corresponding residues or nucleotides. The matrices were used to assess whether the addition of the dsRBD affected the predicted relative arrangement of the dCas13d, crRNA, and target RNA components.

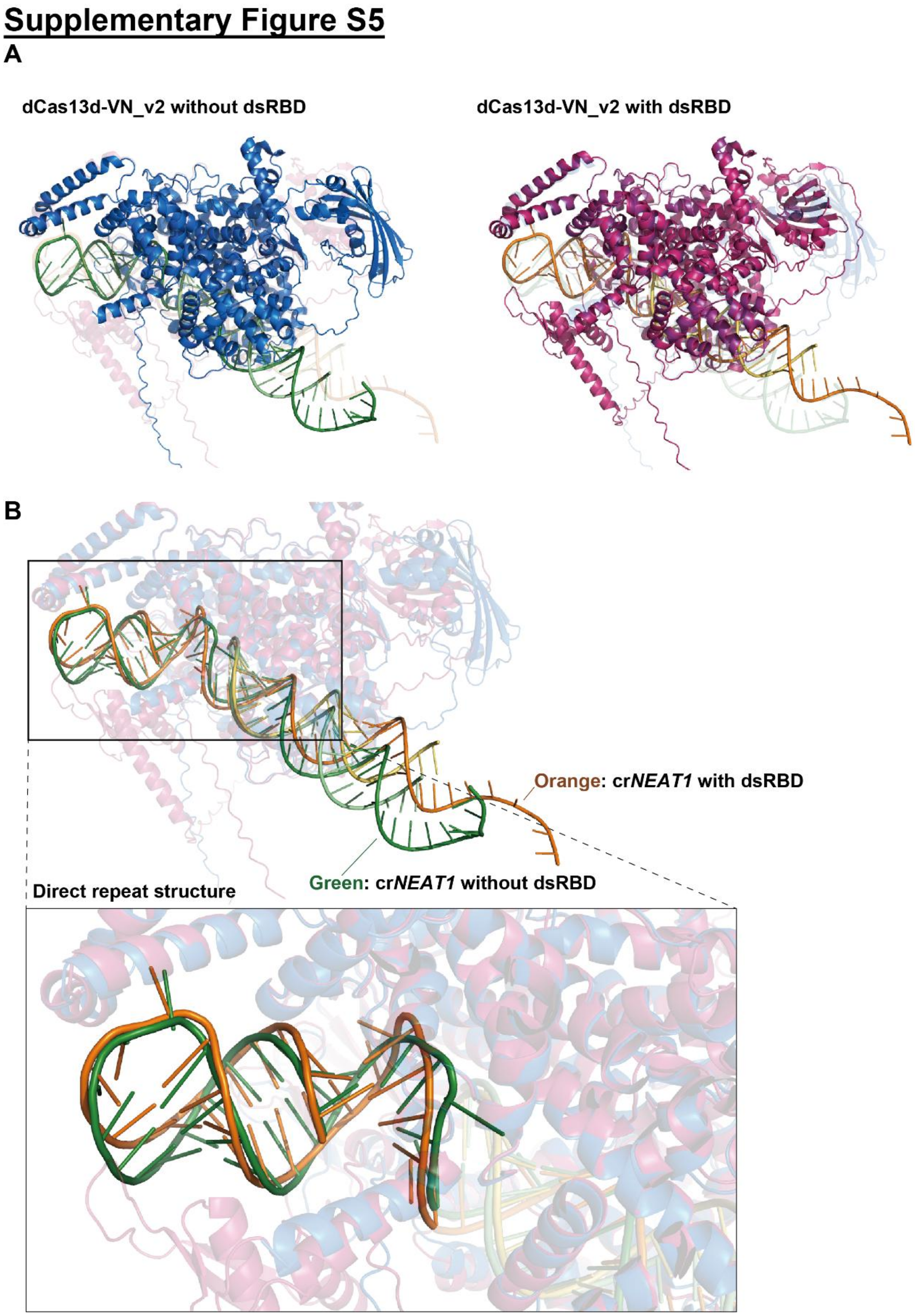

Supplementary Figure S5 (related to Figure 1). Structural comparison of AlphaFold3-predicted BiFC-dCas13d–crRNA–target RNA complexes with or without the appended dsRBD.

A. AlphaFold3-predicted structures of the BiFC-dCas13d_v2-cr*NEAT1*-target RNA complex without the dsRBD (left, blue) and with the appended dsRBD (right, red). Protein and RNA structures were visualized using PyMOL.

B. Structural superposition of the predicted complexes shown in panel A, highlighting the crRNA–target RNA duplex. The crRNA–target RNA duplex without the dsRBD is shown in green, whereas the duplex with the dsRBD is shown in orange. The stem region of the crRNA and its interaction with the target RNA show a high degree of structural overlap between the two models, suggesting that the addition of the dsRBD is not predicted to substantially perturb the overall dCas13d–crRNA–target RNA architecture.

Supplementary Figure S6

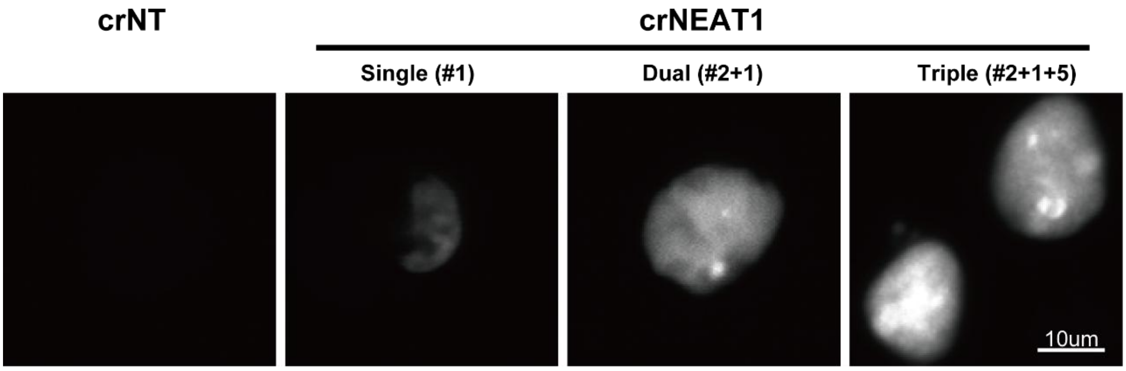

Supplementary Figure S6 (related to Figure 2). Triple cr*NEAT1* shows the highest signal intensity in the BiFC-dCas13 system.

Live-cell imaging of the BiFC-dCas13d_v2 system combined with crNT, single-cr*NEAT1*, dual-cr*NEAT1*, and triple-cr*NEAT1*. 293T cells were transfected with BiFC-dCas13d_v2 components and crRNAs. mVenus fluorescence images were acquired 2 days after transfection using the Nikon A1 confocal microscopic imaging system. Representative mVenus fluorescence images of crNT (left), single-cr*NEAT1* (middle left), dual-cr*NEAT1* (middle right), and triple-cr*NEAT1* (right) are shown. Scale bars: 10 µm.

Supplementary Figure S7

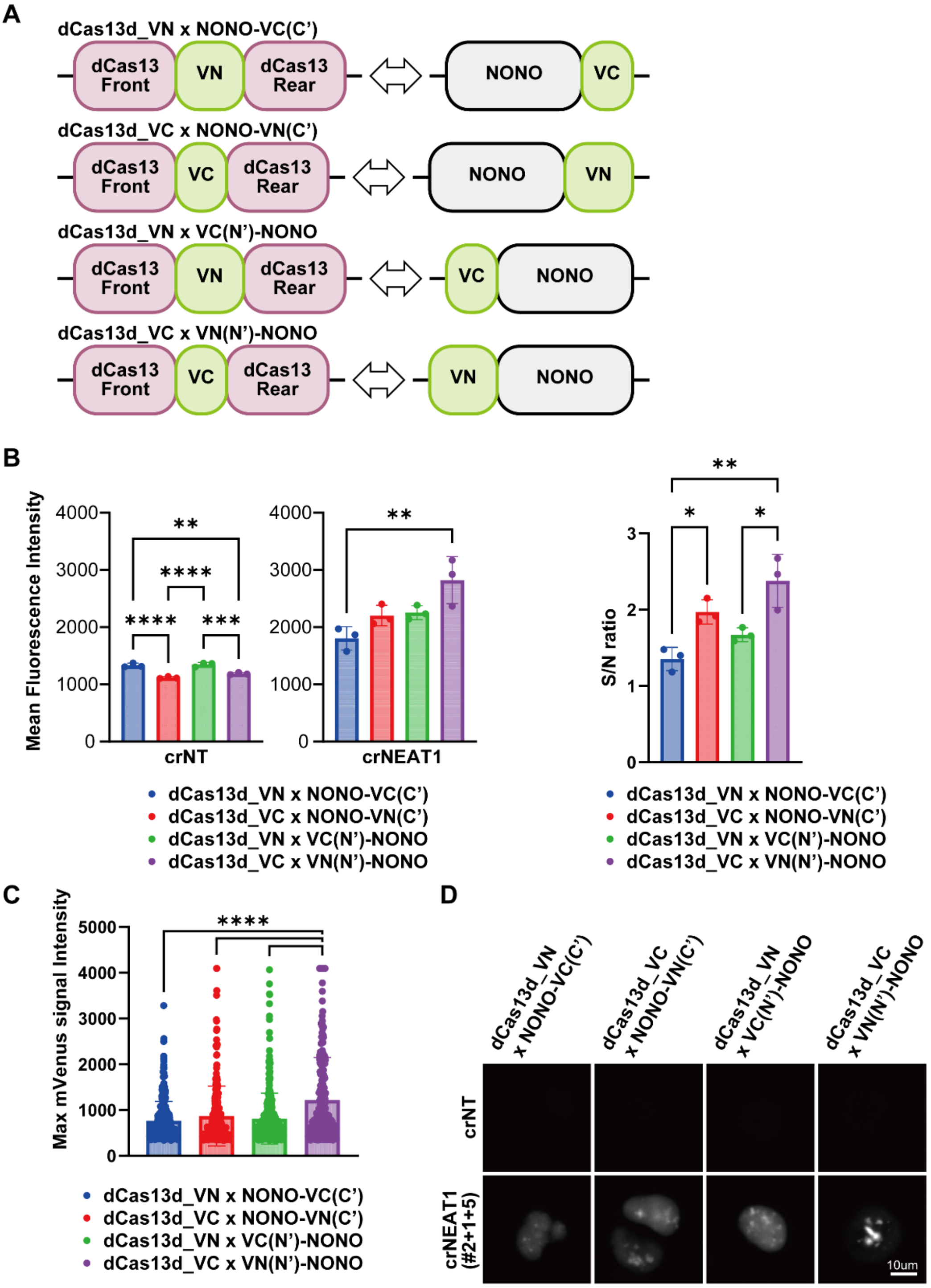

Supplementary Figure S7. Development of BiFC-dCas13d_v3 system.

A. Schematics of the BiFC-dCas13d/NONO combination optimization are shown. For dCas13d, VN155 or VC155 was placed in the middle of the dCas13d sequence. For NONO, VN155 or VC155 was fused at the N- or C-terminus.

B. Mean fluorescence intensities (left) and signal-to-noise (S/N) ratios (right) of four different BiFC-dCas13d configurations are shown as individual data points and the mean +- SD. 293T cells were transfected with crRNAs together with the following combinations of BiFC-dCas13d components: dCas13d-VN155_v2 and NONO-VC155 (NONO-VC155(C')), dCas13d-VC155_v2 and NONO-VN155 (NONO-VN155(C')), dCas13d-VN155_v2 and VC155-NONO (VC155(N')-NONO), or dCas13d-VC155_v2 and VN155-NONO (VN155(N')-NONO). mVenus fluorescence was measured by flow cytometry 2 days after transfection. S/N ratios were calculated by dividing the mean fluorescence intensity (MFI) of the cr*NEAT1* group by that of the crNT group for each BiFC-dCas13d configuration. n = 3 for each group. NT, non-targeting crRNA; Du, dual-cr*NEAT1*; Tri, triple-cr*NEAT1*.

C. Live-cell imaging analysis of the four different combinations of the BiFC-dCas13d system with crNT or triple-cr*NEAT1*. 293T cells were transfected with crRNAs and BiFC-dCas13d components in different combinations. The mVenus fluorescence images were acquired 2 days after transfection using the Nikon A1 confocal microscopic imaging system. Maximum mVenus signal intensities are shown as individual data points and the mean +- SDs. Signal intensities were measured from 293, 268, 366, and 264 cells, respectively. ****$P < 0.0001$ (one-way ANOVA with Tukey-Kramer post hoc test). n = 1.

D. Representative mVenus fluorescence images of the four different combinations of the BiFC-dCas13d system with crNT and triple-cr*NEAT1* (a tandem crRNA construct containing cr*NEAT1*#2, cr*NEAT1*#1, and cr*NEAT1*#5) are shown. Scale bars: 10 μm.

Supplementary Figure S8

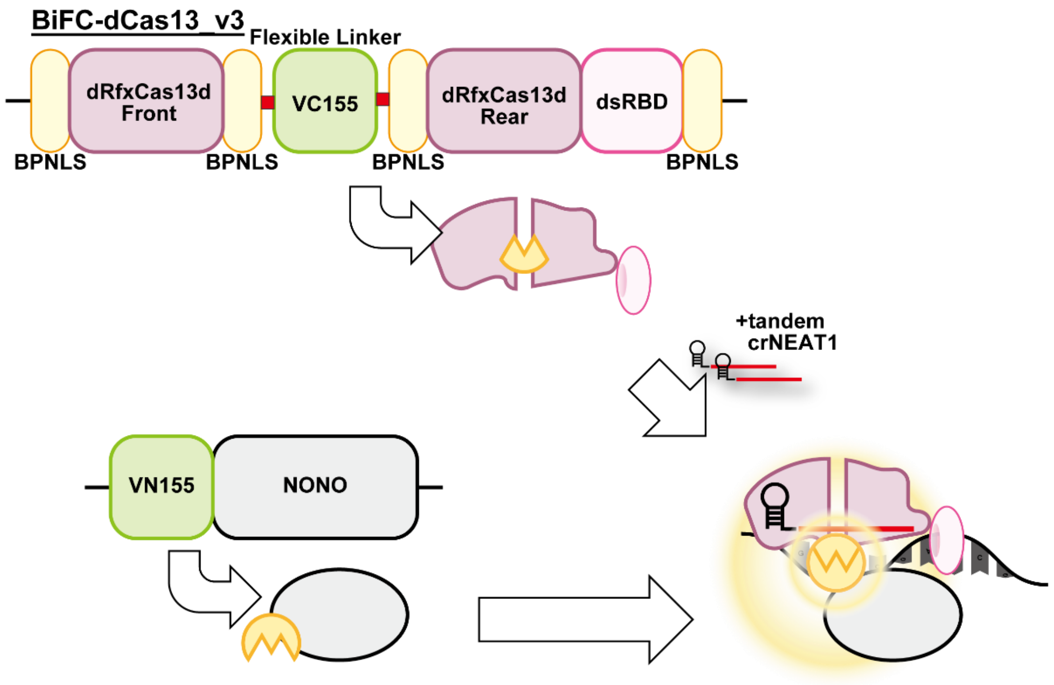

Supplementary Figure S8. BiFC-dCas13d_v3 system.

A schematic of the BiFC-dCas13_v3 system, which is a combination of dCas13-VC155, VN155-NONO, and tandem cr*NEAT1*.

Supplementary Figure S9

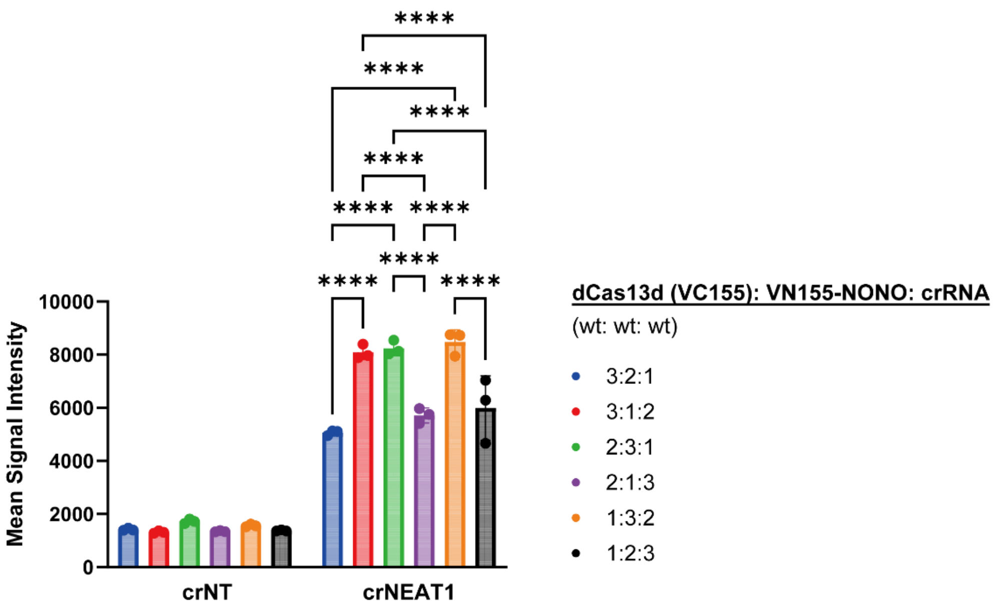

Supplementary Figure S9. Stoichiometric verification of the BiFC-dCas13d_v3 system. The mean fluorescence intensity of the stoichiometrically varied BiFC-dCas13d_v3 system is shown as single data points and the mean +- SD. 293T cells were transfected with BiFC-dCas13d_3 components at different molecular amounts as indicated. The mVenus fluorescence signal was measured 2 days after transfection by flow cytometry. ****P < 0.0001 (one-way ANOVA with Tukey-Kramer post hoc test). n = 3 for each group.

Supplementary Figure S10

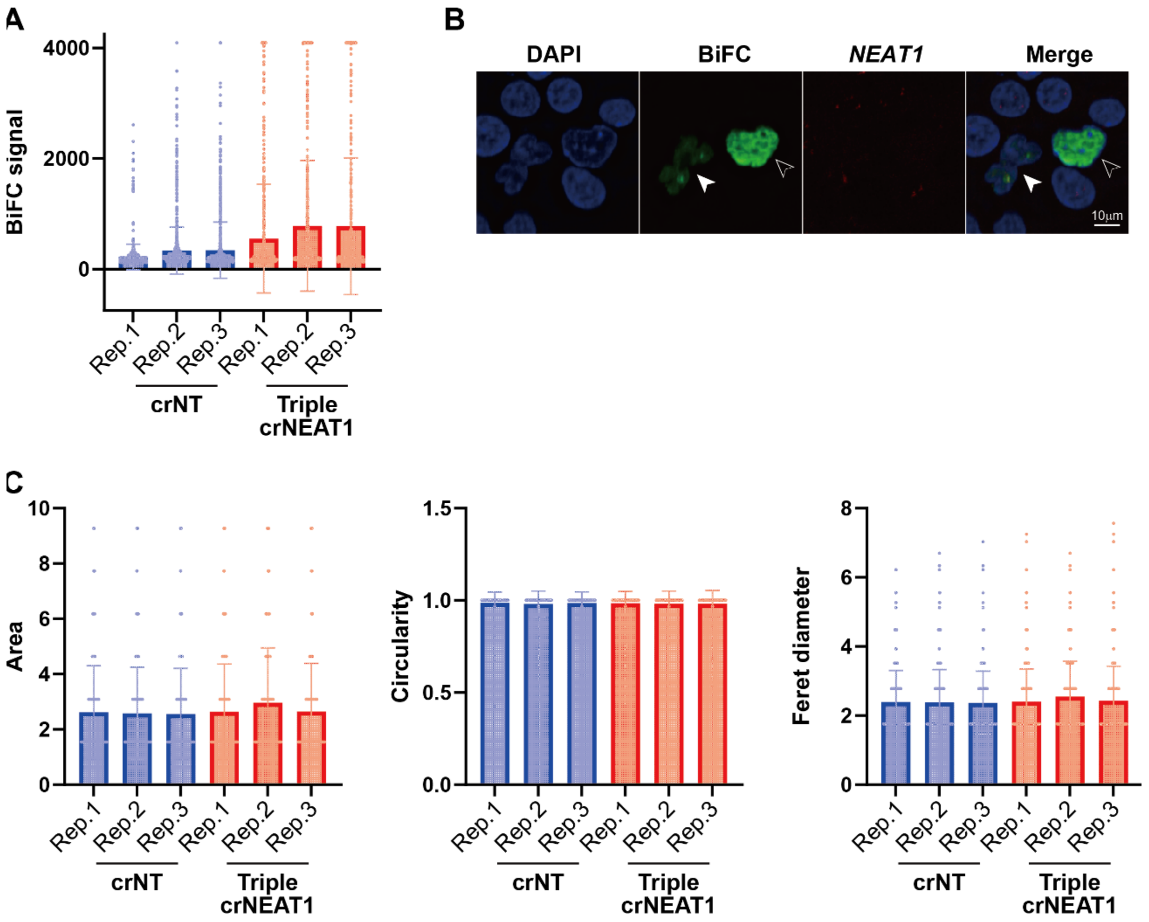

Supplementary Figure S10 (related to Figure 3). Additional quantitative analyses supporting BiFC-dCas13-mediated recording of *NEAT1*–NONO interaction.

A. Quantification of BiFC fluorescence intensity within *NEAT1*-positive regions identified by RNA FISH in three independent biological replicates (Rep. 1–3). 293T cells expressing the optimized BiFC-dCas13 system together with crNT or triple-cr*NEAT1* were fixed 2 days after transfection and subjected to *NEAT1* RNA FISH. Images were acquired from four independent fields per biological replicate, comprising 891–1,282 cells per replicate.

B. Representative fluorescence images of 293T cells expressing the optimized BiFC-dCas13 system together with triple-cr*NEAT1*. White arrowhead indicates cell with punctate BiFC signals overlapping with *NEAT1*-positive foci, whereas black show arrowhead indicates with broader nuclear BiFC signals extending beyond *NEAT1*-positive foci. Scale bars: 10 µm.

C. Quantitative analysis of *NEAT1* RNA FISH foci morphology in three independent biological replicates. The area, circularity, and Feret diameter of *NEAT1*-positive foci are shown for each replicate.

Supplementary Figure S11

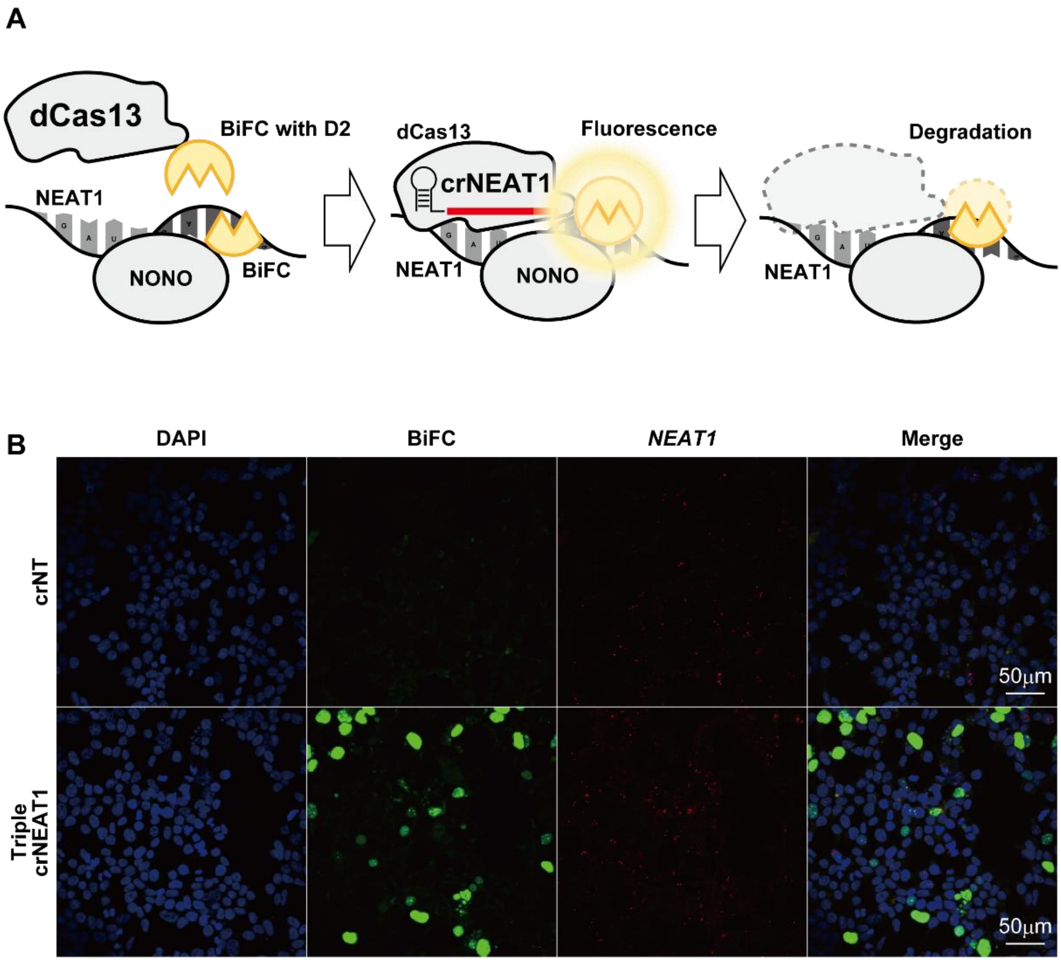

Supplementary Figure S11 (related to Figure 3). Evaluation of a D2-destabilized BiFC-dCas13 system.

A. Schematic illustration of the D2-destabilized BiFC-dCas13 design. A D2 destabilization domain was fused to the BiFC fluorophore to reduce fluorophore persistence following BiFC reconstitution.

B. Representative fluorescence images of 293T cells expressing the D2-BiFC-dCas13 system together with crNT or triple-cr*NEAT1*. Cells were fixed 2 days after transfection and subjected to *NEAT1* RNA FISH. BiFC signals (green), *NEAT1* signals (red), and DAPI (blue) are shown. Scale bars: 50 μm.

Supplementary Figure S12

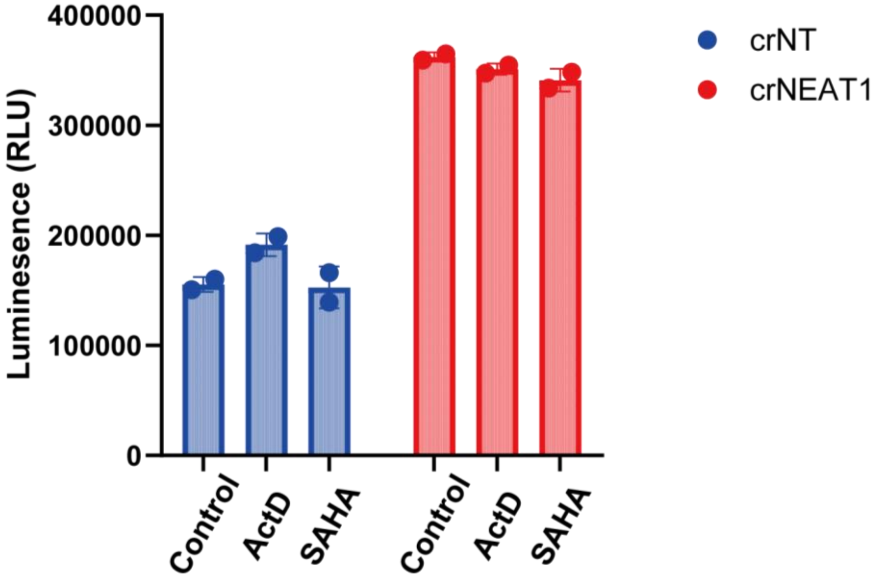

Supplementary Figure S12 (related to Figure 4). Reporter assay using NanoBiT-dCas13 under the drug stimulation.

The luminescence intensity of the NanoBiT-dCas13 systems with crRNAs under the condition of actinomycin D (ActD) and the histone deacetylase inhibitor SAHA is shown as individual data points and the mean +- SD. 293T cells were transfected with NanoBiT-dCas13 components and crRNAs. Two days after transfection, cells were treated with 1 µg/ml of ActD or 5 mM of SAHA for 30 minutes and washed out. Nano-Glo luciferin substrate was added, and luminescence was measured using a microplate reader. n = 2 for each group. Unpaired *t*-test.

Supplementary Figure S13

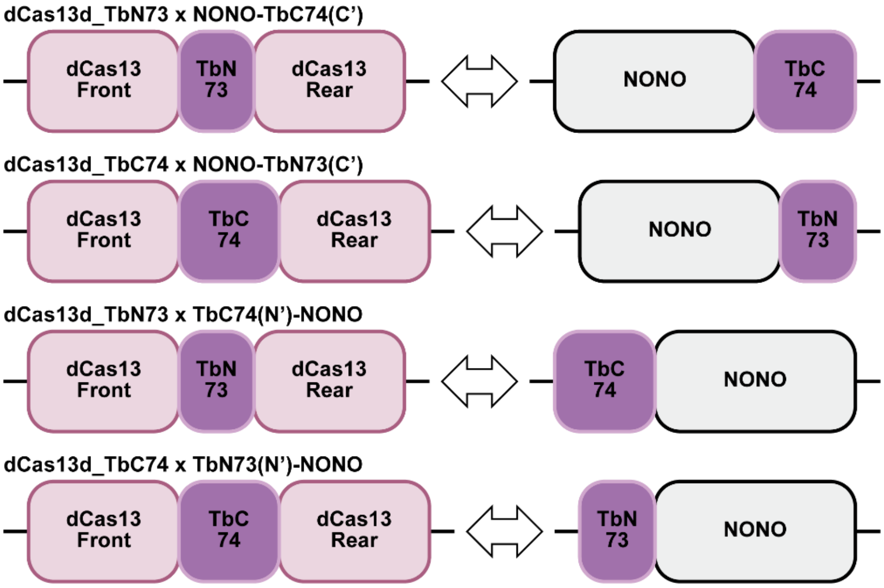

Supplementary Figure S13 (related to Figure 5). Split-TurboID-dCas13d/NONO optimization.

A schematic of the Split-TurboID-dCas13d/NONO combination optimization is shown. For dCas13d, TbN73 or TbC74 was placed in the middle of the dCas13d sequence. For NONO, TbN73 or TbC74 was fused at the N- or C-terminus.

Supplementary Figure S14

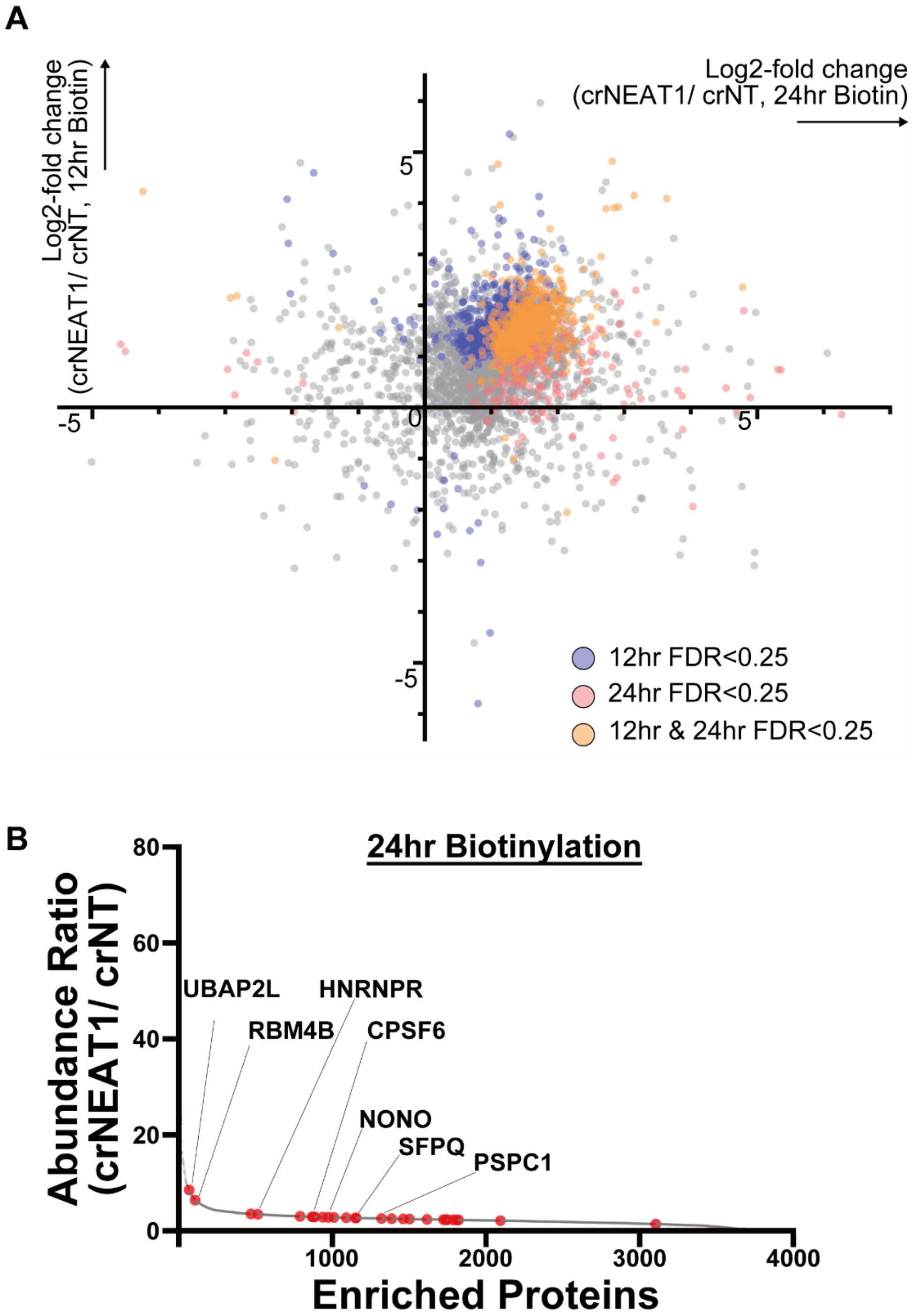

Supplementary Figure S14 (related to Figure 5). LC-MS/MS analysis of biotinylated proteins induced by the split-TurboID dCas13 system.

A. A scatter plot showing the log2-fold change (cr*NEAT1* / crNT, 24-hour biotinylation) versus the log2-fold change (cr*NEAT1*/ crNT, 12-hour biotinylation) for each protein. Blue, red, and

orange dots indicate proteins showing statistical significance (FDR < 0.25) in 12-hour biotinylation, 24-hour biotinylation, and both, respectively.

B. Fold change (cr*NEAT1* / crNT) in protein abundance. Affinity-purified proteins after 24 hours of biotinylation induced by the split-TurboID-dCas13 systems were subjected to LC-MS/MS analysis. Red dots indicate proteins known as paraspeckle components. n = 2 for each group.

Supplementary Figure S15

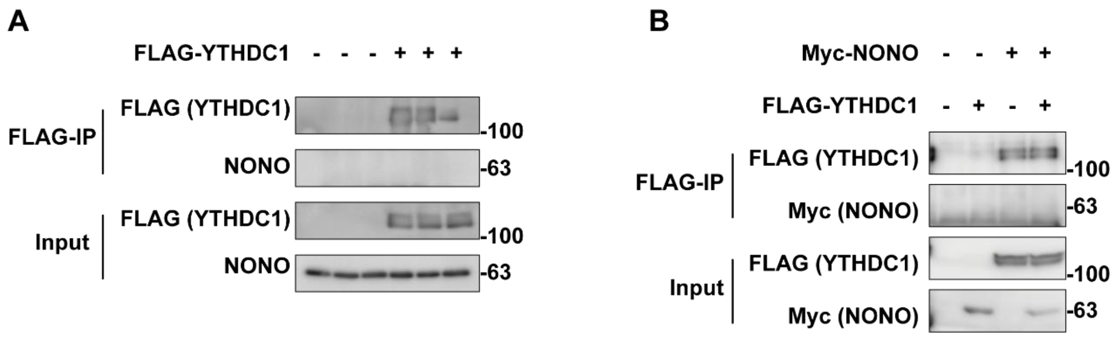

Supplementary Figure S15 (related to Figure 6). Validation of YTHDC1 RIP and co-immunoprecipitation analyses.

A. Validation of FLAG-YTHDC1 expression and immunoprecipitation efficiency in the YTHDC1 RIP assay. 293T cells were transfected with a mock vector or FLAG-YTHDC1. Nuclear lysates were subjected to anti-FLAG immunoprecipitation followed by immunoblotting with the indicated antibodies.

B. Co-immunoprecipitation analysis of YTHDC1 and NONO. 293T cells were transfected with FLAG-YTHDC1 and/or Myc-NONO. Total cell lysates were subjected to anti-FLAG immunoprecipitation followed by SDS-PAGE and immunoblotting with the indicated antibodies.

Supplementary Figure S16

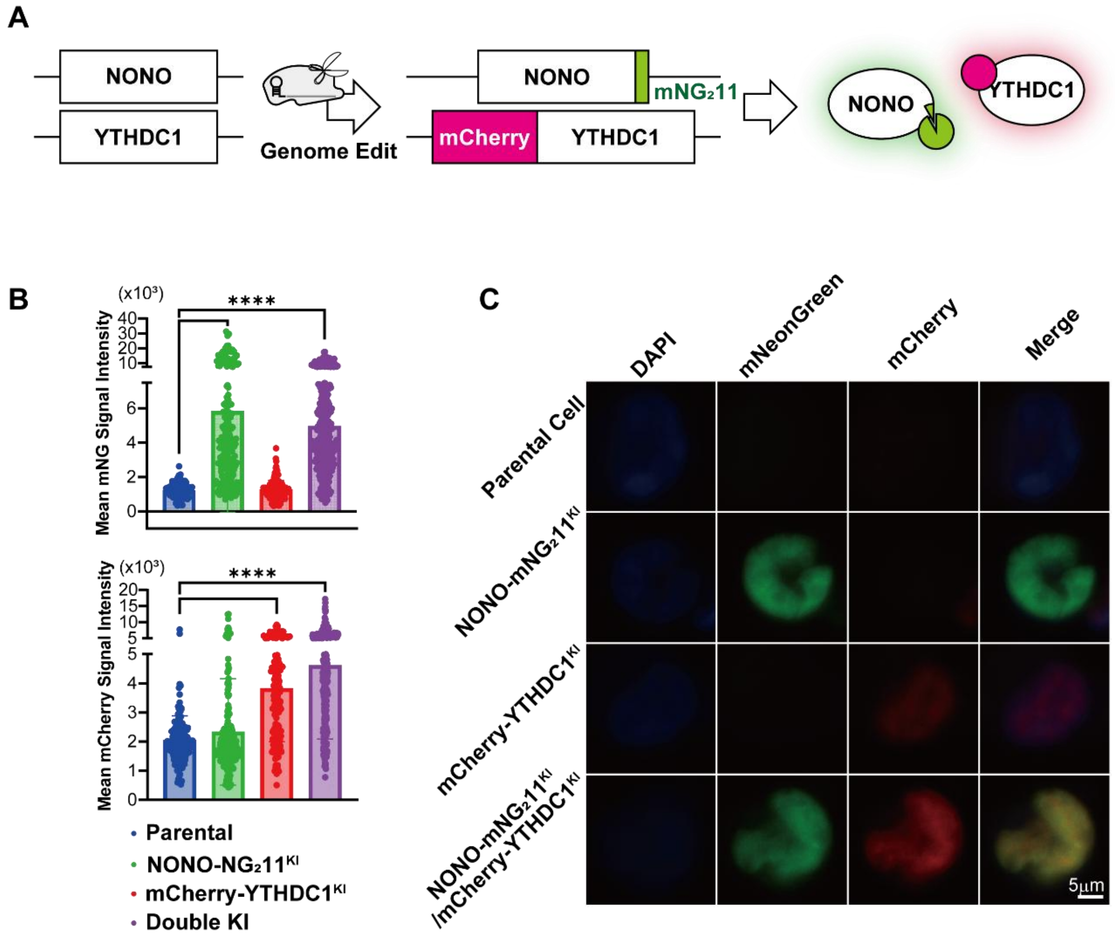

Supplementary Figure S16 (related to Figure 6). Spatial relationship between YTHDC1 and NONO in K562 reporter cells.

A. A schematic of gene editing in K562 cells. K562 cells expressing mNG3A (1-10) were subjected to genome editing to generate NONO-mNeonGreen (mNG) and mCherry-YTHDC1 knock-in cells. $mNG_2 11$ and mCherry protein-coding DNA sequences were introduced directly into the C-terminus of NONO and the N-terminus of YTHDC1, respectively, using CRISPR/Cas9.

B. The mean signal intensities of mNG (left) and mCherry (right) in $mNG_2 11$, mCherry, and dual knock-in cells are shown as individual data points and the mean +- SDs. Gene-edited K562 cells were seeded on glass-bottom dishes one day prior to observation. Fluorescence images were acquired using the Nikon A1 confocal microscopic imaging system. Signal intensities were measured from 174, 203, 168, and 322 cells, respectively. ****$P < 0.0001$ (one-way ANOVA with Dunnett post hoc test).

C. Representative images of parental, and mNG11/ mCherry knock-in cells are shown. Scale bars: 5 μm.

Supplementary Figure S17

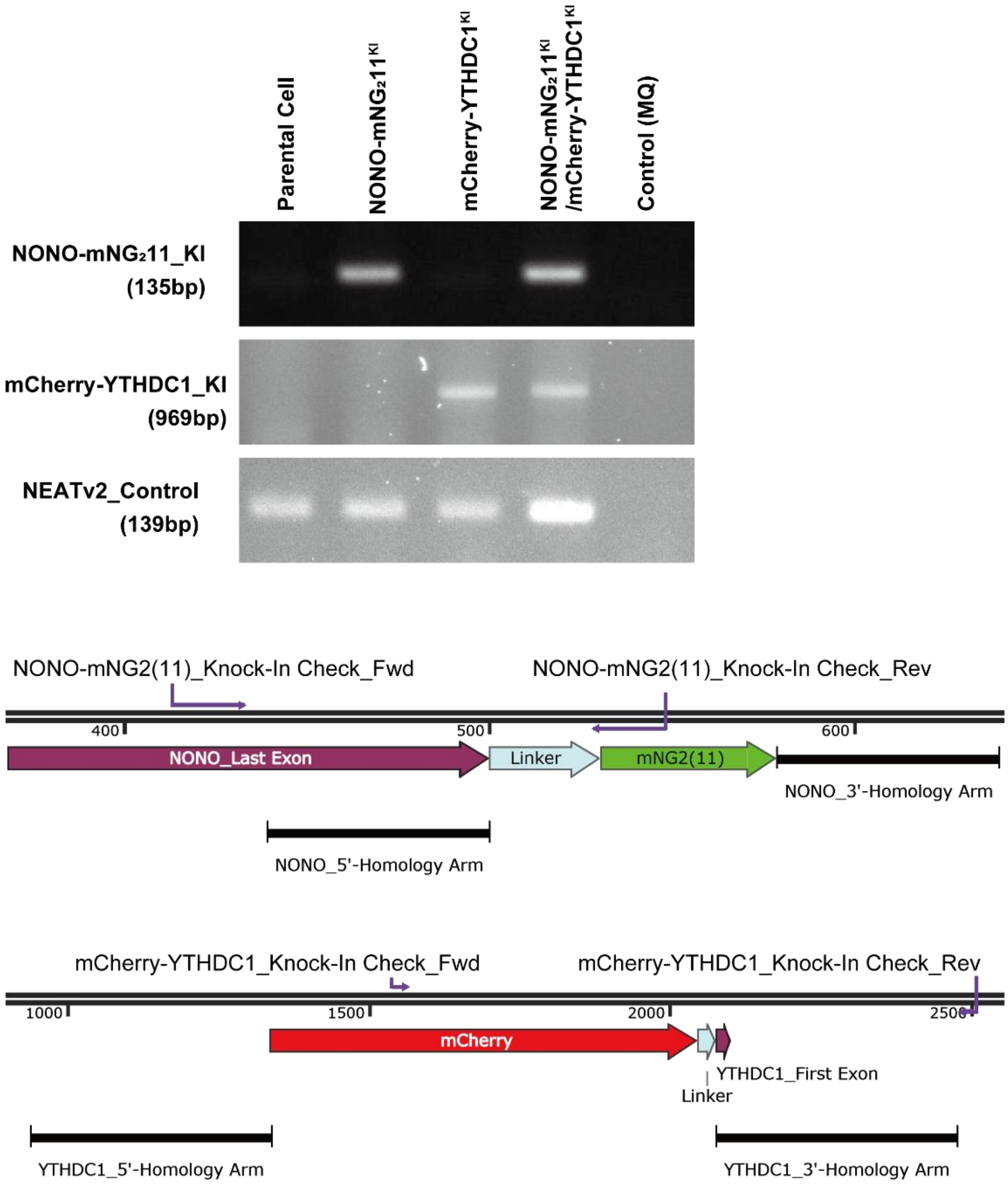

Supplementary Figure S17 (related to Figure 6). Knock-in validation of K562 reporter cells. Confirmation of gene editing in K562/Cas9 cells is shown. DNA was extracted from mNG$_{11}$, mCherry and mNG$_2$11/mCherry dual knock-in cells. The sequences of NONO-mNG$_2$11, mCherry-YTHDC1, and *NEAT1* (control) were amplified by PCR using template DNA from edited cells or water (top). Schematics of the knock-in alleles of NONO-mNG$_2$11 and mCherry-YTHDC1 and the primers used for verification are shown (bottom). One of the primers targeted the knock-in fragment, while the other annealed to the flanking genomic region outside the donor sequence.

Supplementary Figure S18

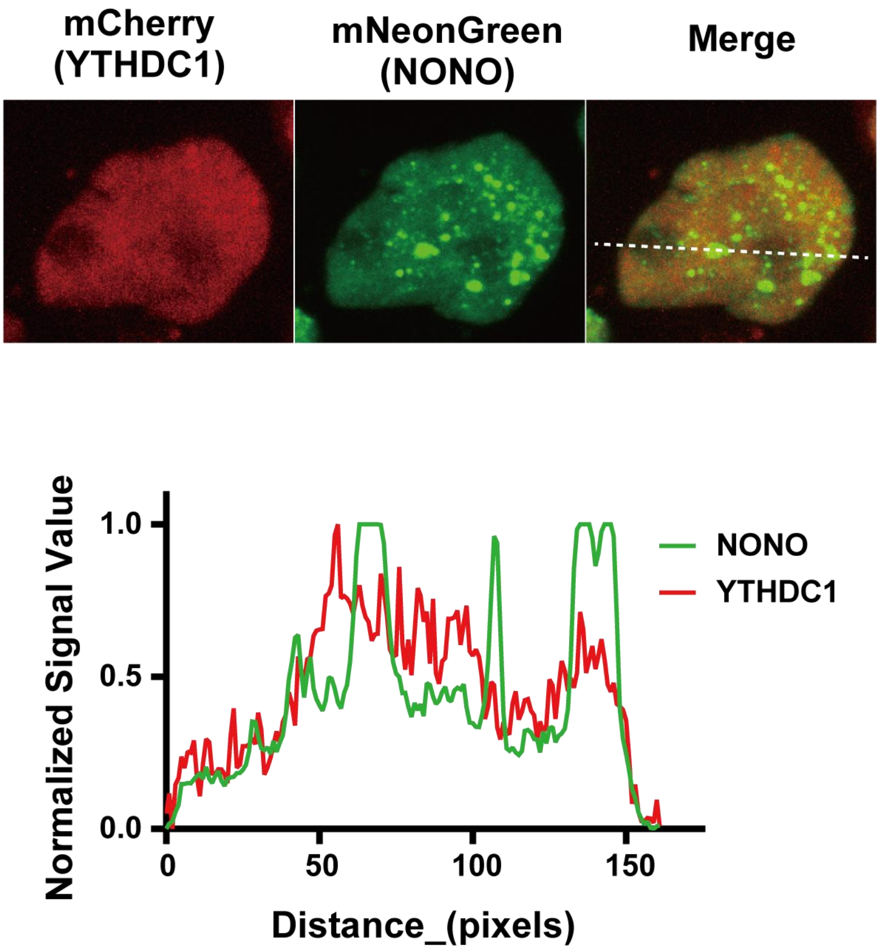

Supplementary Figure S18 (related to Figure 6). Live cell image of mNG11, mCherry, and dual knock-in K562 cells.

Cells were seeded on glass-bottom dishes one day prior to observation. Fluorescence images were acquired using an Olympus IX73 microscope equipped with a CSU-W1 spinning disk confocal unit. Representative images are shown (top). Scale bars: 10 µm. A plot profile analysis of signal intensity after min-max normalization is shown (bottom). Signal intensities of mCherry (red) and mNeonGreen (green) were measured along the dotted lines shown in the representative images.

Supplementary Figure S19

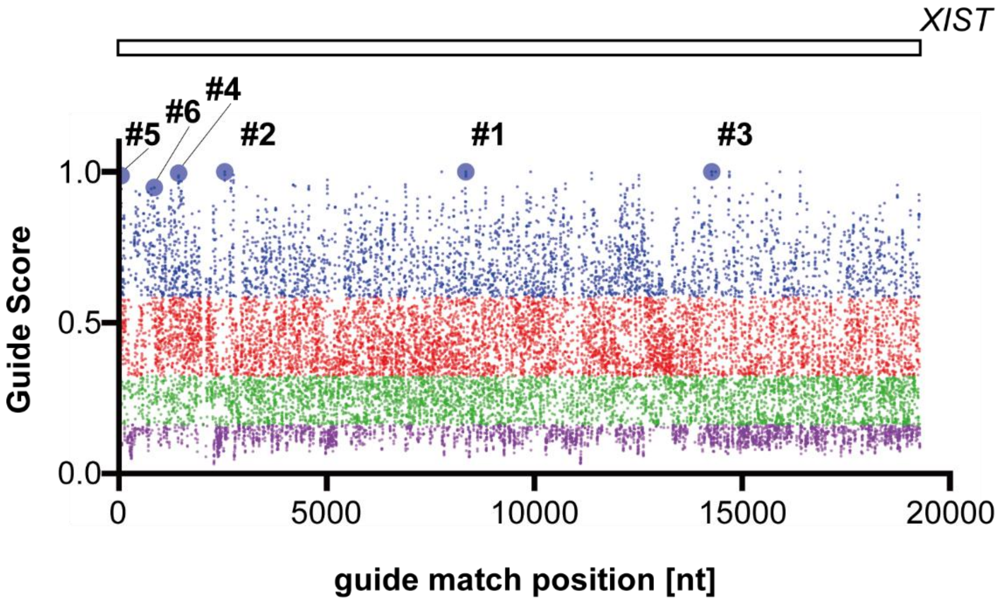

Supplementary Figure S19 (related to Figure 7). Design of crRNAs targeting *XIST*. Guide scores of crRNAs targeting different positions across the *XIST* transcript are shown. Guide scores were calculated using Cas13design (https://cas13design.nygenome.org/). All possible 23-nt crRNAs were designed across the full-length *XIST* transcript and scored according to the Cas13design algorithm. Large circles indicate the crRNAs selected for experimental validation.

Supplementary Figure S20

Feature	BiFC-dCas13	NanoBiT-dCas13	Split-TurboID-dCas13
Tool signature	Fluorescence complementation	Luminescence complementation	Proximity biotinylation
Primary use	Accumulation-based interaction detection	Dynamic interaction monitoring	Proximity interactome screening
Workflow compatibility	Standard fluorescence workflows (FACS / microscopy)	Quantitative luminescence assays	Biotin-streptavidin biochemical assays (IP/ MS)
Signal reversibility	No	Yes	Potentially reversible assembly[38]/ irreversible labeling
Spatial interpretation	Limited by signal persistence	Specialized luminescence imaging required	Proximity-based
Major limitation	Irreversible signal accumulation	Limited compatibility with standard fluorescence-based workflows	Broad/historical proximity labeling due to high enzymatic activity
Best application	Interaction-enriched fluorescence detection	Dynamic perturbation studies	Candidate discovery
**Conceptual role**	**Record**	**Monitor**	**Discover**

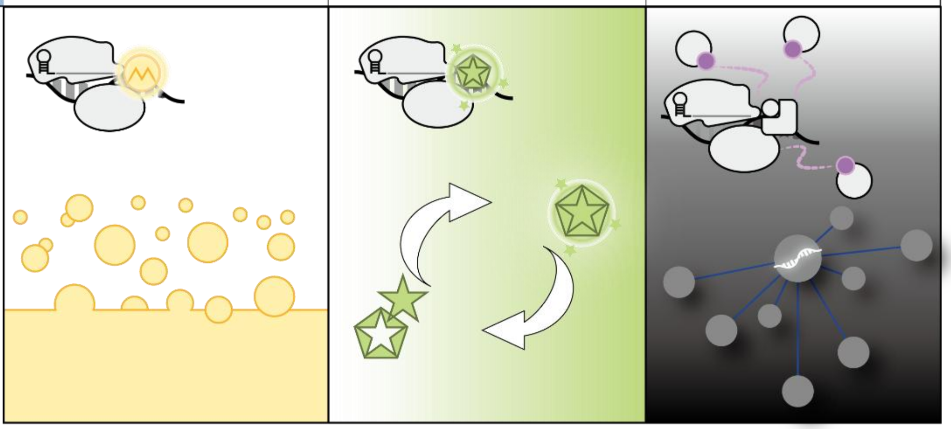

Supplementary Figure S20. Comparative overview of the BiFC-dCas13, NanoBiT-dCas13, and Split-TurboID-dCas13 platforms.
The three dCas13-based RNA–protein interaction analysis platforms developed in this study are compared with respect to their signal-generation principle, primary function, workflow compatibility, signal reversibility, spatial interpretability, major limitations, and recommended applications. BiFC-dCas13 functions as an accumulative fluorescence-based recorder of RNA–protein proximity events, NanoBiT-dCas13 enables reversible monitoring of dynamic interaction changes, and Split-TurboID-dCas13 serves as a proximity-labeling platform for candidate discovery within RPI-associated molecular environments. The lower schematics illustrate the conceptual roles of the three platforms as a recorder, monitor, and discoverer, respectively.
